# Quantitative approximate definable choices

**DOI:** 10.1007/s00208-025-03128-3

**Published:** 2025-03-09

**Authors:** Antonio Lerario, Luca Rizzi, Daniele Tiberio

**Affiliations:** SISSA, via Bonomea 265, 34136 Trieste, Italy

## Abstract

In semialgebraic geometry, projections play a prominent role. A *definable choice* is a semialgebraic selection of one point in every fiber of a projection. Definable choices exist by semialgebraic triviality, but their complexity depends exponentially on the number of variables. By allowing the selection to be approximate (in the Hausdorff sense), we improve on this result. In particular, we construct an approximate selection whose degree is linear in the complexity of the projection and does not depend on the number of variables. This work is motivated by infinite–dimensional applications, in particular to the Sard conjecture in sub-Riemannian geometry. To prove these results, we develop a general quantitative theory for Hausdorff approximations in semialgebraic geometry, which has independent interest.

## Introduction

The goal of this paper is to prove a quantitative approximate version of the *definable choice* theorem, [12, Thm. 4.10]. Before stating our main results, let us explain the name and the context of results of this type.

Let $S\subset \mathbb {R}^{n}$ be a compact semialgebraic set, presented as

1 $$\begin{aligned} S=\bigcup _{i=1}^a\bigcap _{j=1}^{b_i}\left\{ x\in \mathbb {R}^{n}\mid \textrm{sign}(p_{ij}(x))=\sigma _{ij}\right\} , \end{aligned}$$

where $\sigma _{ij}\in \{0, +1, -1\}$, the $p_{ij}$ are polynomials of degree at most *d*. We condense this information into the *diagram* $D(S):=(n, c, d)$ of the representation (1), where $c=a\max b_i$.

We denote by

$$\begin{aligned}\pi :\mathbb {R}^{n}\rightarrow \mathbb {R}^\ell \end{aligned}$$

the linear projection onto the last $\ell$ coordinates. It follows from Tarski–Seidenberg that the set $\pi (S)$ is semialgebraic and a natural problem is to choose, for every element $y\in \pi (S)$, an element $x(y)\in \pi ^{-1}(y)\cap S$ so that the resulting set $A:=\{x(y)\}_{y\in \pi (S)}$ is semialgebraic and of dimension at most $\ell$. The set *A* is called a *definable choice* over $\pi (S)$.

The fact that this can be done follows from a result in semialgebraic geometry called *semialgebraic triviality* (see Corollary 10). However, as a result of this process there is no good control on the geometry of *A* in terms of the data defining *S* (see Remark 11).

For many geometric applications one does not really need that *A* is a choice over $\pi (S)$, but it is enough that it is close to it. More precisely, given $\epsilon >0$, denote by $\mathcal {U}_\epsilon (S)$ the Euclidean $\epsilon$–neighbourhood of *S*. Then one can relax the requirements for the definable choice and ask for a set $A_\epsilon \subseteq \mathcal {U}_\epsilon (S)$, with $\pi (A_\epsilon )$ “close” to $\pi (S)$ (in the Hausdorff metric, denoted by $\textrm{dist}_H$), and possibly with a control on the diagram of $A_\epsilon$.

In this direction we prove two related results. The first one deals specifically with the problem that we have just discussed (see Theorem 44).

### Theorem A

(Quantitative approximate definable choice, first version) For every $c \in \mathbb {N}$ there exist $\kappa \in \mathbb {N}$ such that the following holds. Let $n,\ell ,d\in \mathbb {N}$, with $1\le \ell \le n$. Let $\pi :\mathbb {R}^{n}\rightarrow \mathbb {R}^\ell$ be the projection onto the last $\ell$ coordinates and let $S\subset \mathbb {R}^{n}$ be a bounded closed semialgebraic set with

$$\begin{aligned} D(S)=(n, c, d). \end{aligned}$$

Then, for every $\epsilon >0$ there exists a closed semialgebraic set $A_\epsilon \subset \mathbb {R}^{n}$ such that:

(i) $$\dim (A_\epsilon )\le \ell $$;
(ii) $$A_\epsilon \subseteq \mathcal {U}_\epsilon (S)$$;
(iii) $$\textrm{dist}_{H}(\pi (A_\epsilon ), \pi (S))\le \epsilon $$;
(iv) $$D(A_\epsilon )=(n, \kappa , \kappa d)$$.

The set $A_\epsilon$ from the statement is therefore an approximate (in the Hausdorff metric) definable choice over $\pi (S)$, with a quantitative control on its diagram.

In view of applications, it is also useful to have the following alternative version of the previous result (Theorem 45), which essentially follows from Theorem A applied to the case of the graph of a semialgebraic map.

### Theorem B

(Quantitative approximate definable choice, second version) For every $c, d, \ell \in \mathbb {N}$ there exists $\beta >1$ satisfying the following statement. Let $n\in \mathbb {N}$ and let $K\subset \mathbb {R}^n$ be a closed semialgebraic set contained in the ball $B_{\mathbb {R}^n}(\rho )$, for some $\rho >0$, and let $F:\mathbb {R}^n\rightarrow \mathbb {R}^\ell$ be a locally Lipschitz semialgebraic map such that

$$\begin{aligned} D(\textrm{graph}(F|_K)) = (n+\ell ,c,d). \end{aligned}$$

Then for every $\epsilon \in (0, \rho )$ there exists a closed semialgebraic set $C_\epsilon \subset \mathbb {R}^{n}$ such that:

(i) $$\dim (C_\epsilon )\le \ell $$;
(ii) $$C_\epsilon \subseteq \mathcal {U}_\epsilon (K)$$;
(iii) $$\textrm{dist}_{H}(F(C_\epsilon ), F(K))\le L(F, \rho ) \cdot \epsilon $$, where $$L(F, \rho ):=2+\textrm{Lip}(F, B_{\mathbb {R}^n}(2 \rho ))$$;
(iv) for every $e=1, \ldots , n$ and every affine space $\mathbb {R}^e\simeq E\subseteq \mathbb {R}^n$, the number of connected components of $E\cap C_\epsilon$ is bounded by
  2 $$\begin{aligned} b_0(E\cap C_{\epsilon })\le \beta ^e. \end{aligned}$$

### Remark 1

(The case of polynomial maps) Theorem 45 can be applied to any polynomial map $F:\mathbb {R}^n \rightarrow \mathbb {R}^\ell$ with components of degree bounded by *d*, assuming that the diagram of the original set satisfies $D(K) = (n,c,d)$. In fact, in this case

$$\begin{aligned} \textrm{graph}(F|_K) = \big \{ (x,y)\in \mathbb {R}^n\times \mathbb {R}^\ell \mid x\in K, \, y=F(x)\big \} \end{aligned}$$

is a bounded and closed semialgebraic set with $D(\textrm{graph}(F|_K))=(n+\ell ,c+1,d)$.

Theorem B corresponds to [12, Thm. 4.10 and Ex. 4.11], where it is proved the existence of a semialgebraic set $C_\epsilon$ as in Theorem B, except for (2), which for them has the shape

$$\begin{aligned}b_0(E\cap C_{\epsilon })\le f(n,\ell , c, d, e),\end{aligned}$$

for some (non–explicit) function $f:\mathbb {N}^5\rightarrow \mathbb {N}$. The most notable conclusion from Theorem B is therefore the explicit dependence of the bound on the dimension of the affine space of Item (iv), which says that we can take $f(n, \ell , c, d, e)=\beta (c, d, \ell )^e$.

Similarly, the difficult part from Theorem A is proving that the diagram of $A_\epsilon$ has the explicit shape $D(A_\epsilon )=(m, \kappa , \kappa d)$ with $\kappa$ depending only on the combinatorial data *c* of the diagram of the original set (and not on the number of variables, for instance).

Obtaining this explicit dependence is non–trivial. Compared to [12, Thm. 4.11], a conceptual novelty is the use of ideas from [2], which in turn involves the study of approximation of semialgebraic sets in the Hausdorff metric. A large part of the paper is devoted to expanding and developing these ideas, see Sect. 2.3.

### Hausdorff approximations

Section 2.3 contains new ideas that have their own interest and that are based on the observation that the Hausdorff distance between semialgebraic sets in $\mathbb {R}^n$ can be studied within the framework of semialgebraic geometry. In particular, this notion can be defined also on a real closed extension *R* of the real numbers (see Sect. 2.1), leading to a notion of “Hausdorff distance” between semialgebraic sets in $R^n$. The main result from Sect. 2.3 is then a technique to produce Hausdorff approximations of semialgebraic sets using infinitesimals, i.e. working in the real closed field of algebraic Puiseux series (see Definition 4). In the current paper we allow multiple infinitesimals, which makes the technique handful and practical, but which requires nontrivial extensions of the ideas from [2]. What is important for us is that this technique allows to keep control on the combinatorial part and the degrees of the approximating set: for example, in Proposition 38, we show how to approximate a closed semialgebraic set with a *closed basic* semialgebraic set (see Definition 6) whose combinatorial data and degree are controlled in terms of the diagram of the original set (see the beginning of Sect. 2.4 to appreciate the subtlety of this statement).

### Infinite–dimensional applications

Theorem B was recently used in [8], where we study the Sard property for polynomial maps in *infinite* dimension. The starting point for [8] is a quantitative proof given by Yomdin of the classical Sard’s theorem, in *finite* dimension, that uses ideas from semialgebraic geometry [12].

To explain the key point, recall that for a $C^1$ map $F:\mathbb {R}^n\rightarrow \mathbb {R}^m$, and $\Lambda =(\Lambda _1, \ldots , \Lambda _m)\in \mathbb {R}^m_+$ the set of almost–critical values of *F* is defined by

$$\begin{aligned} C^{\Lambda }(F):= \bigg \{ x \in \mathbb {R}^n\,\bigg | \, \sigma _i(D_x F ) \le \Lambda _i, \quad \forall \, i=1,\dots ,m \bigg \}, \end{aligned}$$

where $\sigma _1(D_x F)\ge \cdots \ge \sigma _m(D_xF)$ are the singular values of $D_xF$ (here we assume $n\ge m$). In the finite–dimensional case, the study of the measure of the critical values of *F* can be reduced to estimating the “size” of the set of its almost critical values, i.e. of the image of its almost critical points. For doing this, one can use the theory of *variations*, introduced by Vitushkin [10] and developed in [12]. (Recall that the *i*-th variation of a set *S*, denoted by $V_i(S)$ is a sort of *i*-dimensional volume of *S*.)

In [12, Cor. 7.4] a quantitative estimate on the variations of the almost–critical values of polynomial maps has been obtained. In that estimate the dimensional parameter *n* does not appear explicitly. Using Theorem B, in [8] we obtain the following result, which makes this dependence explicit.

#### Theorem 2

([8, Theorem G]) Let $n\ge m$, and $p: \mathbb {R}^n \rightarrow \mathbb {R}^m$ be a polynomial map with components of degree $\le d$. For $i= 0, \dots , m$, $\Lambda =(\Lambda _1, \dots , \Lambda _m)\in \mathbb {R}^m_+$ and $\rho >0$, we have

3 $$\begin{aligned} V_i( p ( C^{ \Lambda }( p ) \cap B_{\mathbb {R}^n}(\rho ) )) \le \textrm{cst}(m,\rho )n^m \beta _0^n \Lambda _{0} \cdots \Lambda _{i}, \end{aligned}$$

where $\beta _0=\beta _0(d,m)$ depends only on *d* and *m*, $\textrm{cst}(m,\rho )$ depends only on *m*, *r*, and $\Lambda _0:=1$.

The polynomial dependence on *n* of (3) is a consequence of Item (iv) from Theorem B. Letting $n\rightarrow \infty$ this explicit estimate is what is used in [8] to prove Sard-type results for suitable maps $f:H\rightarrow \mathbb {R}^m$, where *H* is an infinite–dimensional Hilbert space.

## Hausdorff approximations in semialgebraic geometry

### Semialgebraic sets and maps

In order to study Hausdorff approximations of semialgebraic sets in $\mathbb {R}^n$, it will be convenient to work with an extension *R* of the field of real numbers, where real “infinitesimals” will be themselves elements of the field (below we will take for *R* the field of algebraic Puiseux series with coefficients in $\mathbb {R}$). For this reason, in this section we recall the basic notions from semialgebraic geometry over a general *real closed field* and we refer the reader to the monographs [1, 4] for more details.

#### Remark 3

(On the use of the language of real closed fields) The language of real closed fields might be unfamiliar for some readers, but it is especially useful in this context. We want to stress that using this language is not strictly necessary and it is possible that the proofs below can be formulated only using semialgebraic geometry in $\mathbb {R}^n$. However, it has become quite standard in semialgebraic geometry since, once it is introduced, the proofs and the statements become much shorter. Moreover, we are quoting some technical constructions from [1–3] that are stated using this language, and translating them to the classical semialgebraic language would make this part more technical – the precise point where Puiseux series will be used instead of classical real algebraic geometry is Proposition 42. From here, the tools of Hausdorff approximation that we develop in Sect. 2.3 will allow to get back to real sets, keeping control of this process.

Recall that a real closed field *R* is an ordered field whose positive cone is the set of squares of elements from *R*, and such that every polynomial in *R*[*x*] of odd degree has a root in *R*. The order of *R* allows to define the sign of an element $r\in R$ as:

$$\begin{aligned} \textrm{sign}(r):={\left\{ \begin{array}{ll}+1& r>0,\\ -1& r<0,\\ \,\,0& r=0. \end{array}\right. } \end{aligned}$$

We denote by $R_{+}:=\{r\in R\,|\, \textrm{sign}(r)\ge 0\}$ the cone of non-negative elements of *R*.

The field $\mathbb {R}$ is real closed. The main example of real closed field we use is the following.

#### Definition 4

*(Algebraic Puiseux series)* Let *R* be a real closed field and $R(\zeta )$ be the field of rational functions in the variable $\zeta$. The real closed field $R\langle \zeta \rangle$ of *algebraic Puiseux series with coefficients in R* is defined by:

$$\begin{aligned} R\langle \zeta \rangle :=\left\{ f=\sum _{k=k_0}^{\infty } a_k\zeta ^{\frac{k}{q}}\,\bigg |\,a_k\in R,\, k_0\in \mathbb {Z},\,q\in \mathbb {N},\, f\text { is algebraic over }R(\zeta )\right\} . \end{aligned}$$

In order to define a ring structure on the set $R\langle \zeta \rangle$, we insist that for $r_1, r_2\in \mathbb {Q}$ it holds

$$\begin{aligned} \zeta ^{r_1}\zeta ^{r_2}=\zeta ^{r_1+r_2}, \quad (\zeta ^{r_1})^{r_2}=\zeta ^{r_1 r_2},\quad \zeta ^0=1. \end{aligned}$$

Therefore, two Puiseux series $a=\sum _{k\ge k_1}a_k\zeta ^{k/q_1}$ and $b=\sum _{k\ge k_2}b_k\zeta ^{k/q_2}$ can be written as a formal power series in $\zeta ^{1/q}$, where *q* is the least common multiple between $q_1$ and $q_2$. This allows to add and multiply Puiseux series. If $a=a_1\zeta ^{r_1}+a_2\zeta ^{r_2}+\cdots \in R\langle \zeta \rangle ,$ with $a_1\ne 0$ and $r_1<r_2<\cdots$, we say that $a>0$ if $a_1>0.$ From this definition it follows that $0<\zeta <r$ for every $r\in R$ with $r>0$, and this is why $\zeta$ is called *infinitesimal*.

#### Remark 5

(Algebraic Puiseux series as germs) The field $R\langle \zeta \rangle$ is isomorphic, as a real closed field, to the field of continuous semialgebraic functions germs $f:(0, \delta )\rightarrow R$, where $(0, \delta )$ is an interval in *R*, [1, Thm. 3.17]. Addition and multiplication translates to the usual ones. The order is defined as follows: the germ of a semialgebraic function $f:(0, \delta )\rightarrow R$ is positive if and only if there exists $0<\delta '<\delta$ such that $f(\zeta )>0$ for every $\zeta \in (0, \delta ')$. Given a germ of a continuous semialgebraic function $f:(0, \delta )\rightarrow R$, in order to get an expression like $f=\sum _{k\ge k_0}a_k\zeta ^{k/q}$, i.e. the corresponding element in the field of algebraic Puiseux series, one uses the fact that the graph of *f* lies on a *branch* of an algebraic curve $P(x, y)=0$ in the plane $R^2$, where *P* is a polynomial with coefficients in *R*. In particular, this branch admits a parametrization near the origin as

$$\begin{aligned} x(\tau )=\tau ^q,\quad y(\tau )=\sum _{k\ge k_0}a_k \tau ^{k}, \end{aligned}$$

where $q\in \mathbb {N}, k_0\in \mathbb {Z}$ and $a_k\in R$, [11, Thm. 2.2]. This means that $P(x(\tau ), y(\tau ))=0$ as formal power series, and one gets a Puiseux series for *f* by the substitution $\tau =\zeta ^{1/q}$ in $y(\tau )$.

If *R* is a real closed field, then so is $R\langle \zeta \rangle$, see [1, Cor. 2.98]. Using this observation, letting $K=R\langle \zeta _1\rangle$, then also $R\langle \zeta _1, \zeta _2\rangle =K\langle \zeta _2\rangle$ is a real closed field. Repeating this argument inductively, we define the real closed field of *algebraic Puiseux series with multiple infinitesimals and coefficients in R* as

$$\begin{aligned}R\langle \zeta _1, \ldots , \zeta _m\rangle :=R\langle \zeta _1\rangle \cdots \langle \zeta _m\rangle . \end{aligned}$$

Let *R* be a real closed field. The *norm* of an element $x=(x_1, \ldots , x_n)\in R^n$ is defined as

$$\begin{aligned}\Vert x\Vert :=\sqrt{x_1^2+\cdots +x_n^2}\in R.\end{aligned}$$

For every $x=(x_1, \ldots , x_n)\in R^n$ and $r\in R_{+}$, we denote by ${B}_{R^n}(x,r)\subset R^n$ the set

$$\begin{aligned} B_{R^n}(x,r):=\left\{ y\in R^n\,\bigg |\, \Vert x-y\Vert \le r\right\} , \end{aligned}$$

and call it the *closed ball around* *x* *of radius* *r*. The *Euclidean topology* on $R^n$ is the topology generated by the closed balls. These notions are inspired by their classical counterparts in the case $R=\mathbb {R}$, with the caveat that in these definitions *r* is not necessary real. Similarly, the order of a real closed field *R* can be used to define the notion of semialgebraic sets.

#### Definition 6

(Semialgebraic sets and maps) We say that a set $S\subset R^n$ is *semialgebraic* if

4 $$\begin{aligned} S=\bigcup _{i=1}^a\bigcap _{j=1}^{b_i}\left\{ x\in R^n\mid \textrm{sign}(p_{ij}(x))=\sigma _{ij}\right\} , \end{aligned}$$

for some finite set of polynomials $p_{ij}\in R[x_1, \ldots , x_n]$ and $\sigma _{ij}\in \{0, +1, -1\}.$ We call the description (4) a *representation* of *S*.

Given two sets of polynomials $\mathcal {P}=\{p_1, \ldots , p_{a_1}\},\mathcal {Q}=\{q_1, \ldots , q_{a_2}\}\subset R[x_1, \ldots , x_n]$, we define the *algebraic* set $Z(\mathcal {P};R)$ and the *closed basic* semialgebraic set $\textrm{Bas}(\mathcal {P}, \mathcal {Q};R)\subset R^n$ by

$$\begin{aligned}  &   Z(\mathcal {P};R):=\{x\in R^n\mid p_1(x)=\cdots =p_{a_1}(x)=0\},\\  &   \textrm{Bas}(\mathcal {P}, \mathcal {Q}; R):=Z(\mathcal {P};R)\cap \left( \bigcap _{j=1}^{a_2}\left\{ x\in R^n\mid q_j(x)\le 0\right\} \right) . \end{aligned}$$

Finally, a map $f:A\rightarrow B$ between semialgebraic sets $A\subset R^n, B\subset R^\ell$ is said to be *semialgebraic* if its graph is a semialgebraic set in $R^n\times R^\ell$.

The representation of a semialgebraic set *S* as in (4) is not unique, however having such a representation quantifies the complexity of *S*, using the following notion.

#### Definition 7

(Diagram of a semialgebraic set) Let $S\subset R^n$ be a semialgebraic set represented as in (4). We say that the triple

$$\begin{aligned} \left( n, a\cdot \max _i\{{b_i}\}, \max _{i,j}\{\deg (p_{ij})\}\right) \in \mathbb {N}^3 \end{aligned}$$

is a *diagram* for *S*. Below, the equation “$D(S)= (m, c, d)$” will mean that there exists a representation of *S* as in (4) with $n\le m$, $a\cdot \max _i\{{b_i}\}\le c$ and $\max _{i,j}\{\deg (p_{ij})\}\le d$.

It is often useful to use an alternative (equivalent) description of semialgebraic sets, using the notion of *first–order formulas*.

#### Definition 8

(First-order formula) Let *R* be a real closed field. A first-order formula of the language of ordered fields with coefficients in *R* is a formula written with a finite number of conjunctions, disjunctions, negations, and universal or existential quantifiers on variables, starting from atomic formulas which are formulas of the kind $p(x_1, \ldots , x_n) = 0$ or $q(x_1, \ldots ,x_n) < 0$, where *p* and *q* are polynomials with coefficients in *R*. The free variables of a formula are those variables of the polynomials appearing in the formula which are not quantified.

Semialgebraic sets are precisely those defined by first-order formulas [4, Prop. 2.2.4].

#### Definition 9

(Set defined by a formula, see)[1, Sect. 1.1] Let $\psi$ be a first-order formula of the language of ordered fields, with coefficients in *R*, and with *n* free variables. The *set defined by* $\psi$ in $R^n$ (or the *realization* of $\psi$ in $R^n$) is the semialgebraic set

$$\begin{aligned} \textrm{Reali}(\psi ; R)\subseteq R^n \end{aligned}$$

defined by induction on the construction of the formula, starting from atoms:

$$\begin{aligned}\textrm{Reali}(p =0;R):=\{x\in R^n\,|\, p(x)=0\},\quad \textrm{Reali}(p< 0;R):= \{x \in R^n \,|\, p(x)< 0\},\end{aligned}$$

(*p* is a polynomial with coefficients in *R*),

$$\begin{aligned}  &   \textrm{Reali}(\phi _1 \wedge \phi _2; R):= \textrm{Reali}(\phi _1; R) \,\cap \, \textrm{Reali}(\phi _2; R),\\  &   \textrm{Reali}(\phi _1 \vee \phi _2; R):= \textrm{Reali}(\phi _1; R)\, \cup \, \textrm{Reali}(\phi _2; R),\\  &   \qquad \qquad \textrm{Reali}( \lnot \phi ; R ):= R^n \setminus \textrm{Reali}(\phi ; R ),\\  &   \textrm{Reali}((\exists y) \,\phi ; R):= \{x\in R^n \,|\, \exists y\in R,\, (x, y) \in \textrm{Reali}(\phi ; R)\},\\  &   \textrm{Reali}((\forall y)\, \phi ; R):= \{x \in R^n\,|\, \forall y\in R,\, (x, y) \in \textrm{Reali}(\phi ;R)\}, \end{aligned}$$

where $\phi _1, \phi _2, \phi$ are first-order formulas with an appropriate number of free variables.

### Some properties of semialgebraic sets

We collect here some properties of semialgebraic sets over a real closed field.

#### Semialgebraic triviality

Continuous semialgebraic maps $f:A\rightarrow B$ are “piecewise” trivial fibrations: there exists a partition of *B* into finitely many semialgebraic sets

$$\begin{aligned} B=\bigsqcup _{j=1}^b B_j \end{aligned}$$

and, for every $j=1, \ldots , b$ there exist fibers $F_j:=f^{-1}(y_j)$, for some $y_j\in B_j,$ and a semialgebraic homemorphism $\varphi _j:B_j\times F_j\rightarrow f^{-1}(B_j)$ that makes the following diagram commutative:This result is called *semialgebraic triviality*, see [4, Thm. 9.3.2].

##### Corollary 10

Definable choices exist.

##### Proof

Let $S\subset \mathbb {R}^{n}$ be a semialgebraic set and $\pi :\mathbb {R}^n\rightarrow \mathbb {R}^\ell$ the projection. Then, by semialgebraic triviality there exists a finite partition of $\pi (S)=\sqcup _{j=1}^b B_j$ into semialgebraic sets and for every $j=1, \ldots , b$ there exists fibers $F_j=\pi ^{-1}(y_j)\subset S$, with $y_j\in B_j$, and semialgebraic homeomorphisms $\varphi _j:B_j\times F_j\rightarrow \pi ^{-1}(B_j)\cap S$ making the corresponding diagram commutative. For every $j=1, \ldots , b$ choose an element $x_j\in F_j$. The definable choice of *S* over $\pi (S)$ is the set

$$\begin{aligned} \bigcup _{j=1}^b\varphi _j \left( B_j \times \{x_j\} \right) , \end{aligned}$$

concluding the proof. $\square$

##### Remark 11

Note that the complexity of a set built as in the proof of Corollary 10 depends on the complexity of the objects involved in the semialgebraic triviality. This uses the so–called cylindrical algebraic decomposition and it is known to be doubly exponential in the number of variables of *S* (see [5, 6]).

#### Dimension and stratifications

Every semialgebraic subset of $R^n$ can be written as a finite union of semialgebraic sets, each of them semialgebraically homeomorphic to an open cube $(0,1)^m\subset R^m$, for some $m \le n$ ([4, Thm. 2.3.6]). This allows to define the *dimension* of a semialgebraic set as the maximum of the dimensions *m* of these cubes. In fact, it is possible to introduce the notion of smoothness also over general real closed fields, and every semialgebraic set can be written as a finite union of smooth, semialgebraic disjoint manifolds called *strata* ([4, Prop. 9.1.8]). This is called a *Nash stratification*. We will use this last result only in the classical case $R=\mathbb {R}.$

The dimension of a semialgebraic set is preserved by semialgebraic homeomorphisms and behaves naturally under Cartesian product:

$$\begin{aligned}\dim (A\times B)=\dim (A)+\dim (B).\end{aligned}$$

Moreover, if $f:A\rightarrow B$ is a continuous semialgebraic map, then

5 $$\begin{aligned} \dim (f(A))\le \dim (A). \end{aligned}$$

We will also need a stronger notion of dimension, introduced in [3].

##### Definition 12

(Strong dimension) Let $S\subset R^{n}$ be a semialgebraic set and $1\le \ell \le n$. We say that *S* is *strongly of dimension* $\le \ell$ if, letting $\pi _\ell : R^{n} \rightarrow R^\ell$ be the projection on the last $\ell$ coordinates, it holds

6 $$\begin{aligned} \forall y\in R^\ell \quad \#\left( S \cap \pi _{\ell }^{-1}(y)\right) <\infty . \end{aligned}$$

It follows from semialgebraic triviality (see Sect. 2.2.1) that if (6) holds, then

7 $$\begin{aligned} \sup _{y\in R^\ell } \#\left( S \cap \pi _{\ell }^{-1}(y)\right) <\infty , \end{aligned}$$

so that in Definition 12 one can replace (6) with the apparently stronger condition (7).

Moreover, if *S* is strongly of dimension $\le \ell$, then $\dim (S)\le \ell$. However, a semialgebraic set *S* of dimension $\ell$ may not be strongly of dimension $\le \ell$, as this depends on the relative position between *S* and the projecting subspace $R^\ell$. Nevertheless we have the following result.

##### Proposition 13

(Genericity of strong dimension) Let $S\subset R^{n}$ be a semialgebraic set of dimension $\ell$. The set of invertible linear transformations $L:R^{n}\rightarrow R^{n}$ such that *L*(*S*) is strongly of dimension $\le \ell$ is semialgebraic and dense.

##### Proof

We first prove the case $R=\mathbb {R}$. The set of such linear transformations is semialgebraic, as it admits a semialgebraic description in the coefficients of *L*.

To prove density, let $M\subset \mathbb {R}^{n}$ be a smooth *p*-dimensional manifold with $p\le \ell$. Given $r\in \mathbb {N}$ and a Thom–Boardman manifold $\Sigma \subset J^{r}(M, \mathbb {R}^\ell )$, it follows from [9, Thm. 2] that the set of linear maps $T:\mathbb {R}^{n}\rightarrow \mathbb {R}^\ell$ that restrict to maps $T|_M:M\rightarrow \mathbb {R}^\ell$ which are transversal to $\Sigma$ is dense. In particular, the set of linear maps $T:\mathbb {R}^{n}\rightarrow \mathbb {R}^\ell$ such that $T|_{M}$ is a stratified immersion is dense. The fibers of every such map are discrete and every such map can be obtained as $T=\pi _\ell \circ L$ for an appropriate $L:\mathbb {R}^{n}\rightarrow \mathbb {R}^{n}$. Therefore, for every smooth *p*-dimensional manifold $M\subset \mathbb {R}^{n}$, with $p\le \ell$, the set ${\mathcal {L}}(M)$ of linear maps $L:\mathbb {R}^{n}\rightarrow \mathbb {R}^{n}$ such that the fibers of the map $\pi _\ell |_{L(M)}:L(M)\rightarrow \mathbb {R}^\ell$ are discrete is dense. When *M* is semialgebraic, the set ${\mathcal {L}}(M)$ is also semialgebraic and therefore it contains an open dense set.

Let $S=\sqcup _{i=1}^a M_i$ be a Nash stratification of *S*. Applying the above argument to each stratum $M_i$, we see that the set ${\mathcal {L}}(S):={\mathcal {L}}(M_1)\cap \cdots \cap {\mathcal {L}}(M_a)$ is semialgebraic and contains an open dense set. For every $L\in {\mathcal {L}}(S)$ the fibers of $\pi _\ell |_{L(S)}:L(S)\rightarrow \mathbb {R}^{\ell }$ are discrete and semialgebraic, therefore they are finite. Hence *L*(*S*) is strongly of dimension $\le \ell$. This concludes the proof for the case $R=\mathbb {R}$.

The statement we just proved can be written as a first-order formula with coefficients in $\mathbb {R}$. Therefore, the same statement holds if $\mathbb {R}$ is replaced by a real closed extension *R* of it, by [4, Prop. 5.2.3]. (This is the so-called *transfer principle* from real algebraic geometry: every property that can be expressed in the first-order language of ordered fields with coefficients in *R* can be transferred to any real closed extension of *R*.) $\square$

#### Thom-Milnor bound

For every $c\in \mathbb {N}$ there exists $\beta _{\textrm{tm}}=\beta _{\textrm{tm}}(c)>1$ such that, if *S* is a semialgebraic set with diagram $D(S)=(n, c, d)$, then the number of connected components of *S*, denoted by $b_0(S)$, can be bounded by ([1, Thm. 7.50]):

8 $$\begin{aligned} b_0(S)\le \beta _{\textrm{tm}}d^n. \end{aligned}$$

### Hausdorff approximations using infinitesimals

Using Puiseux series we can produce Hausdorff approximations of semialgebraic sets in $\mathbb {R}^n$ as “specializations” of semialgebraic sets defined on an extension $R^n$ which contains some “infinitesimals”, and this can be done in a controlled way. Let us explain this idea.

#### Example 14

Given a set $A\subset \mathbb {R}^n$, for $r>0$ denote its *r*–neighbourhood in $\mathbb {R}^n$ by

$$\begin{aligned} \mathcal {U}_r(A):=\left\{ x\in \mathbb {R}^n\,\bigg |\, \exists a=(a_1, \ldots , a_n)\in A \quad \text {s.t.} \quad \sum _{i=1}^n(a_i-x_i)^2 \le r^2\right\} . \end{aligned}$$

If *A* is semialgebraic, defined by a first order formula $\phi$, then $\mathcal {U}_r(A)$ is defined by the first order formula

$$\begin{aligned} \phi _r:=\left( \exists a \left( \phi (a) \wedge \sum _{i=1}^n(a_i-x_i)^2\le r^2\right) \right) . \end{aligned}$$

Instead of interpreting $\{\phi _r(x)\}_{r>0}$ as a *family* of first order formulas with coefficients in $\mathbb {R}$, we can interpret it as a single first order formula $\psi$ with coefficients in $R=\mathbb {R}\langle \zeta \rangle$

$$\begin{aligned} \psi :=\left( \exists a \left( \phi (a) \wedge \sum _{i=1}^n(a_i-x_i)^2\le \zeta ^2\right) \right) . \end{aligned}$$

The set $S\subset R^n$ defined by the formula $\psi$ encodes the family of neighbourhoods of the original semialgebraic set *A* in a semialgebraic way. Note that the *r*–neighbourhood of *A* is described by the formula obtained from *S* by “evaluating” the infinitesimal $\zeta$ at *r*.

#### The evaluation map and the map ${\mathscr {L}}_0$

The procedure described in Example 14 is a special case of a general construction of “evaluation” of a semialgebraic set of Puiseux series. First, we provide the following definition, inspired by [7, Def. 1.7], to formalize the concept of properties that are true for “sufficiently small” nested sequences of Puiseux series. The intricacy of the statements for the case $m>1$ is a consequence of the iterative definition of Puiseux series $R\langle \zeta _1,\dots ,\zeta _m\rangle$ for several infinitesimals.

##### Definition 15

(Predicates for sufficiently small Puiseux series) Let *R* be a real closed field, $1\le k\le m$, and let $\mathscr {P}=\mathscr {P}(t_k, \dots , t_m)$ be a property where $t_j\in R\langle \zeta _1, \ldots , \zeta _{j-1}\rangle$ for $k\le j\le m$. We say that the property $\mathscr {P}$ holds for

$$\begin{aligned}0<t_k\ll \cdots \ll t_{m}\ll 1\end{aligned}$$

if there exists $\delta _m\in R\langle \zeta _1, \ldots , \zeta _{m-1}\rangle$ with $\delta _m>0$ such that for $t_m\in R\langle \zeta _1, \ldots , \zeta _{m-1}\rangle$ with $0<t_m<\delta _m$ there exists $\delta _{m-1}\in R\langle \zeta _1, \ldots , \zeta _{m-2}\rangle$ with $\delta _{m-1}>0$ such that for all $t_{m-1}\in R\langle \zeta _1, \ldots , \zeta _{m-2}\rangle$ with $0<t_{m-1}<\delta _{m-1}$ there exists (...) $\delta _k\in R\langle \zeta _1, \ldots , \zeta _{k-1}\rangle$ with $\delta _k>0$ such that for all $t_k\in R\langle \zeta _{1}, \ldots \zeta _{k-1}\rangle$ with $0<t_k<\delta _k$ we have $\mathscr {P}(t_k, \ldots , t_m)$.

##### Definition 16

(The evaluation map) Let *R* be a real closed field and let $\psi$ be a formula with *n* free variables and with coefficients in $R\langle \zeta _1, \ldots , \zeta _m\rangle$. For every $1\le k\le m$ and for every $(t_k, \ldots , t_m)$ with $t_j\in R\langle \zeta _1, \ldots , \zeta _{j-1}\rangle$ for $j=k, \ldots , m$, we denote by $\psi |_{\zeta _k=t_k, \ldots , \zeta _m=t_m}$ the formula with coefficients in $R\langle \zeta _1, \ldots , \zeta _{k-1}\rangle$ that is obtained from $\psi$ by replacing iteratively in its coefficients each $\zeta _j$ by $t_j$, starting from $j=m$. This can be done provided that, at each step, $t_j$ is sufficiently small so that the coefficients of the formula coming from the previous step (which are finitely many and which we see as germs of continuous semialgebraic functions in $\zeta _j$) have a representative defined on a common interval containing $t_j$, yielding a Puiseux series in $j-1$ infinitesimals. If $S\subset R\langle \zeta _1, \ldots , \zeta _m\rangle ^n$ is the semialgebraic set defined by $\psi$, we denote by $S|_{t_k, \ldots , t_m}\subset R\langle \zeta _1, \ldots , \zeta _{k-1}\rangle ^n$ the semialgebraic set defined by the formula $\psi |_{\zeta _k=t_k, \ldots , \zeta _m=t_m}$, i.e.

9 $$\begin{aligned} S|_{t_k, \ldots , t_m}:=\textrm{Reali}\bigg (\psi |_{\zeta _k=t_k, \ldots , \zeta _m=t_m}; R\langle \zeta _1, \ldots , \zeta _{k-1}\rangle \bigg )\subset R\langle \zeta _1, \ldots , \zeta _{k-1}\rangle ^n, \end{aligned}$$

provided that the right hand side of (9) is defined. In particular, this is the case for $0<t_k\ll \dots \ll t_{m}\ll 1$. The set $S|_{t_k, \ldots , t_m}$ is called the *evaluation* of *S* (at $t_k,\dots ,t_m$).

##### Remark 17

Indeed, (9) depends on the formula $\psi$ defining *S*; for us the formula will be clear and this abuse of notation will create no harm.

##### Remark 18

(Polynomial coefficients) If the coefficients of the formula $\psi$ are *polynomials* in the infinitesimals (and not general algebraic Puiseux series), then (9) is defined for any $(t_k,\dots ,t_m)$ with $t_j \in R[\zeta _1,\dots ,\zeta _{j-1}]\subset R\langle \zeta _1,\dots ,\zeta _{j-1}\rangle$. In this case, at the *i*–th step of the process one gets a new formula with coefficients in $R[\zeta _1, \ldots , \zeta _{m+i-2}]$; therefore the process can be iterated and the concept of “sufficiently small” from Definition 15 can be dispensed of. This is the approach followed in [2] in the case of one infinitesimal ($m=1$). Our general formulation, albeit defined only for $0<t_k\ll \dots \ll t_m\ll 1$, is more flexible and technically necessary. Notably, the key Lemmas 21 and 22 require evaluation at general Puiseux series.

##### Remark 19

(Evaluations and germs) Let $S=\textrm{Reali}(\psi ;R\langle \zeta \rangle )\subset R\langle \zeta \rangle ^n$ be a semialgebraic set. Recall from Remark 5 that the germ of a continuous semialgebraic function $g:(0, \delta )\rightarrow R$ can be identified to an element of $R\langle \zeta \rangle$ (we denote this germ still by *g*). Then, using the notation from Definition 16, the following property is true by construction:

10 $$\begin{aligned} \exists \delta >0\quad \forall r\in (0,\delta )\quad g(r)\in S|_r \quad \iff g\in S. \end{aligned}$$

##### Lemma 20

(Evaluation preserves the diagram) In the same setting of Definition 16, for every $1\le k\le m$ it holds

$$\begin{aligned} D(S|_{t_k,\dots ,t_m})= D(S), \end{aligned}$$

for all $(t_k,\dots ,t_m)$ such that the evaluation is defined.

##### Proof

The statement follows from the fact that a representation of $S|_{t_1,\dots ,t_m}$ is obtained from a representation of *S* by replacing $\zeta _j$ with $t_j$ in the coefficients, in the prescribed order. $\square$

The strong dimension condition of Definition 12 is not increased by small evaluations.

##### Lemma 21

(Small evaluations do not increase the strong dimension: one infinitesimal) In the same setting of Definition 16, with $m=1$. Let $\pi _\ell : R\langle \zeta \rangle ^{n}\rightarrow R\langle \zeta \rangle ^\ell$ be the projection on the last $\ell$ coordinates, $1\le \ell \le n$. Then, there exists $\delta \in R$, $\delta >0$ such that for all $t \in R$ with $0<t<\delta$ it holds

$$\begin{aligned} \sup _{y \in R^\ell } \# S|_{t}\cap \pi _\ell ^{-1}(y) \le \sup _{w \in R\langle \zeta \rangle ^\ell } \# S\cap \pi _\ell ^{-1}(w). \end{aligned}$$

In particular, if $S\subset R\langle \zeta \rangle ^n$ is strongly of dimension $\le \ell$, then the set $S|_{t}$ is also strongly of dimension $\le \ell$.

##### Proof

In the proof, set $\pi = \pi _\ell$. Let

11 $$\begin{aligned} N:=\sup _{w\in R\langle \zeta \rangle ^\ell }\#\left( S\cap \pi ^{-1}(w) \right) . \end{aligned}$$

If $N=\infty$ there is nothing to prove, then assume $N<\infty$.

Assume by contradiction that for all $\delta \in R$, $\delta >0$, there exists $t\in R$, $0<t<\delta$, and $y\in R^\ell$ such that

$$\begin{aligned} \#S|_t\cap \pi ^{-1}(y) >N. \end{aligned}$$

Consider the semialgebraic set $D\subset R^\ell \times R_+$ defined by

$$\begin{aligned} D:=\left\{ (y,t)\in R^\ell \times R_+\mid \# S|_t \cap \pi ^{-1}(y) > N\right\} . \end{aligned}$$

Let $P_2: D \rightarrow R_+$ be the projection on the second factor. By our assumption the image $P_2(D)\subset R_+$ contains a sequence accumulating to 0. By semialgebraic triviality (Sect. 2.2.1) there exists $\delta _0\in R_+$, $\delta _0>0$, a point $t_0\in (0,\delta _0)$ a fiber $F_0 = P_2^{-1}(t_0)$ and a semialgebraic homeomorphism $\Phi : (0,\delta _0)\times F_0 \rightarrow P_2^{-1}((0,\delta _0))$ such that the following diagram is commutativeFix an element $f_0\in F_0$ and consider the function $y_0:(0,\delta _0)\rightarrow R^\ell$ given by

$$\begin{aligned} y_0(t):= P_1\circ \Phi (t,f_0), \end{aligned}$$

where $P_1: D \rightarrow R^\ell$ denotes the projection in the first factor. The (components of the) function $y_0$ are semialgebraic and continuous curves on the right of the origin, thus the corresponding germs (which we denote with the same symbol) define algebraic Puiseux series $y_0 \in R\langle \zeta \rangle ^\ell$. Furthermore, we note that by construction $(y_0(t),t) \in D$ for all $t\in (0,\delta _0)$ so that

12 $$\begin{aligned} \forall t\in (0,\delta _0), \qquad \#\left( S|_t\cap \pi ^{-1}(y_0(t))\right) > N. \end{aligned}$$

Denoting by $(v,w)\in R\langle \zeta \rangle ^{n} = R\langle \zeta \rangle ^{n-\ell }\times R\langle \zeta \rangle ^{\ell }$, the set $\pi ^{-1}(y_0(t))\subset R^{n}$ can be seen as the evaluation at *t* of the set $\pi ^{-1}(y_0) \in R\langle \zeta \rangle ^n$, defined by the formula $(w=y_0)$ with variables (*v*, *w*) and coefficients in $R\langle \zeta \rangle$.

Thus for $0<t\ll 1$ (which means, according to Definition 15, up to taking a smaller $\delta _0$, for all $0<t<\delta _0$), it holds

$$\begin{aligned} \left. \left( S\cap \pi ^{-1}(y_0)\right) \right| _t&= \textrm{Reali}\Big ((\psi \cap (w=y_0))|_{\zeta = t};R\Big ) \\&= \textrm{Reali}\Big (\psi |_{\zeta =t};R\Big )\cap \textrm{Reali}\Big ((w=y_0)|_{\zeta =t};R\Big ) \\&= S|_t \cap \pi ^{-1}(y_0(t)). \end{aligned}$$

In particular from (12) we obtain

13 $$\begin{aligned} \forall t\in (0,\delta _0) \qquad \# \left. \left( S\cap \pi ^{-1}(y_0)\right) \right| _t = \#\left( S|_t\cap \pi ^{-1}(y_0(t))\right) > N. \end{aligned}$$

Consider then the semialgebraic set $T\subset R^n\times R_+$ defined by

$$\begin{aligned} T:=\left\{ (z,t)\in R^n\times R_+\,\Big |\, z\in \left. \left( S \cap \pi ^{-1}(y_0)\right) \right| _{t}\right\} . \end{aligned}$$

Denote by $P: T\rightarrow R_+$ the projection on the last factor. By semialgebraic triviality, for $\delta _0>0$ small enough, we find a semialgebraic homeomorphism

$$\begin{aligned} \varphi : (0,\delta _0)\times H \rightarrow P^{-1}((0,\delta _0)), \end{aligned}$$

where $H=P^{-1}(\bar{t})$ for some $\bar{t}\in (0,\delta _0)$. By (13) such a fiber must have cardinality $>N$. Pick $N+1$ distinct points $h_1,\dots ,h_{N+1}\in H$. Denote by $Q:T\rightarrow R^n$ the projection on the first factor and define for $j=1,\dots ,N+1$ continuous semialgebraic curves $g_j:(0,\delta _0)\rightarrow R^n$ by

$$\begin{aligned} g_j(t):=Q(\varphi (t,h_j)). \end{aligned}$$

By construction, these curves satisfy

$$\begin{aligned} \forall t\in (0,\delta _0)\quad g_j(t)\in \left. \left( S\cap \pi ^{-1}(y_0)\right) \right| _{t}. \end{aligned}$$

Therefore (the germ of) each $g_j \in S \cap \pi ^{-1}(y_0)\subset R\langle \zeta \rangle ^n$, by (10). Since the elements $h_j$ are distinct, the germs of these curves near zero represent $N+1$ distinct points in $S\cap \pi ^{-1}(y_0)$, contradicting (11).

We have proved that there exists $\delta >0$ such that for all $t\in R$ with $0<t<\delta$ it holds

$$\begin{aligned} \sup _{y\in R^\ell } \#S|_t\cap \pi ^{-1}(y)\le \sup _{w\in R\langle \zeta \rangle ^\ell } \# S\cap \pi ^{-1}(w). \end{aligned}$$

which is the statement. $\square$

We will need a version of Lemma 21 for multiple infinitesimals ($m>1$).

##### Lemma 22

(Small evaluations do not increase the strong dimension: several infinitesimals) In the same setting of Definition 16, let $\pi _\ell : R\langle \zeta _1,\dots ,\zeta _m\rangle ^{n}\rightarrow R\langle \zeta _1,\dots ,\zeta _m\rangle ^\ell$ be the projection on the last $\ell$ coordinates, $1\le \ell \le n$, and let $1\le k\le m$. Then for $0<t_k\ll \cdots \ll t_m\ll 1$ it holds

$$\begin{aligned} \sup _{y \in R\langle \zeta _1,\dots ,\zeta _{k-1}\rangle ^\ell } \# S|_{t_k,\dots ,t_m}\cap \pi _\ell ^{-1}(y) \le \sup _{w \in R\langle \zeta _1,\dots ,\zeta _{m}\rangle ^\ell } \# S\cap \pi _\ell ^{-1}(w). \end{aligned}$$

In particular, if $S\subset R\langle \zeta _1,\dots ,\zeta _m\rangle ^n$ is strongly of dimension $\le \ell$, then for $0<t_k\ll \cdots \ll t_m\ll 1$ the set $S|_{t_k,\dots ,t_m}$ is also strongly of dimension $\le \ell$.

##### Proof

Note that Lemma 21 corresponds to the case $m=1$. To prove the case $m>1$, recall that $R\langle \zeta _1,\dots ,\zeta _m\rangle \simeq K\langle \zeta \rangle$, with $K:=R\langle \zeta _1,\dots ,\zeta _{m-1}\rangle$. We find $\delta _m\in R\langle \zeta _1,\dots ,\zeta _{m-1}\rangle$, $\delta _m>0$, such that for all $t_m \in R\langle \zeta _1,\dots ,\zeta _{m-1}\rangle$ with $0<t_m<\delta _m$ it holds

$$\begin{aligned} \sup _{y \in R\langle \zeta _1,\dots ,\zeta _{m-1}\rangle ^\ell } \#S|_{t_m}\cap \pi ^{-1}(y) \le \sup _{w\in R\langle \zeta _1,\dots ,\zeta _{m}\rangle ^\ell } S \cap \pi ^{-1}(w). \end{aligned}$$

We can now iterate the argument *k*-times, with $1\le k\le m$ to get the statement, using the definition of $S|_{t_k,\dots ,t_m}$. $\square$

##### Definition 23

(Bounded elements and the limit homomorphism: one infinitesimal)Let *R* be a real closed field. An element $s\in R\langle \zeta \rangle$ is called *bounded over* *R* if $\Vert s\Vert \le r$ for some $r\in R_+$. The subring $R\langle \zeta \rangle _b$ of elements that are bounded over *R* consists of algebraic Puiseux series with non-negative exponents.

For all $n\in \mathbb {N}$, we define the *limit homomorphism*

14 $$\begin{aligned} \lambda _{\zeta }:R\langle \zeta \rangle _b^n\rightarrow R^n,\end{aligned}$$

by the ring homomorphism mapping $\sum _{k\ge 0}^{\infty } a_k\zeta ^{\frac{k}{m}}$ to $a_0\in R^n$.

##### Remark 24

Viewing $R\langle \zeta \rangle$ as the field of germs of semialgebraic functions continuous on the right of zero, the bounded elements corresponds to those germs that have a finite limit as $\zeta \rightarrow 0$ and

$$\begin{aligned}\lambda _\zeta (f)=\lim _{\zeta \rightarrow 0}f(\zeta ).\end{aligned}$$

From this we see that the map $\lambda _\zeta$ is order preserving, in the following sense: if $f_1,f_2\in R\langle \zeta \rangle _b$ with $f_1\le f_2$, then $\lambda _\zeta (f_1)\le \lambda _\zeta (f_2)$.

##### Definition 25

(Bounded elements: several infinitesimals)Let *R* be a real closed field. We say that $f=(f_1, \ldots , f_n)\in R\langle \zeta _1, \ldots , \zeta _m\rangle ^n$ is *bounded over R* if $\Vert f\Vert \le r$ for some $r\in R_{+}$.

##### Remark 26

The subring $R\langle \zeta _1, \ldots , \zeta _m\rangle ^n_b$ of elements that are bounded over *R* contains algebraic Puiseux series with non-negative exponents. However the inclusion is strict: the Puiseux series $t= \zeta _1^{-1}\zeta _2 \in R\langle \zeta _1,\zeta _2\rangle$ is such that $\Vert t\Vert \le r$ for all $r\in R_+$, $r>0$.

Note that if *R* is real closed, then on the set of elements of $R\langle \zeta _1, \ldots , \zeta _m\rangle ^n$ that are bounded over *R* (i.e. with $\Vert f\Vert \le r$ with $r\in R_+$) the composition of maps

$$\begin{aligned} R\langle \zeta _1, \ldots , \zeta _m\rangle _b^n{\mathop {\longrightarrow }\limits ^{\lambda _{\zeta _m}}}R\langle \zeta _1, \ldots , \zeta _{m-1}\rangle _b^n{\mathop {\longrightarrow }\limits ^{\lambda _{\zeta _{m-1}}}}\cdots R\langle \zeta _1\rangle _b^n{\mathop {\longrightarrow }\limits ^{\lambda _{\zeta _1}}}R^n\end{aligned}$$

is well–defined, since at every step we get bounded elements.

##### Remark 27

We stress that the composition above is well–defined only if taken with the order prescribed by the infinitesimals. For instance, let $f\in \mathbb {R}\langle \zeta _1, \zeta _2\rangle _{b}$ be given by

$$\begin{aligned}f=\sum _{k\ge 0}a_k(\zeta _1)\zeta _2^{\frac{k}{q}} \quad \text {where}\quad a_k(\zeta _1)=\sum _{j\ge 0}b_{k,j}\zeta _1^{\frac{j}{q_k}}.\end{aligned}$$

(Note that each $a_k(\zeta _1)$ has “its own” $q_k$.) Then

$$\begin{aligned}f=\sum _{k\ge 0}\left( \sum _{j\ge 0}b_{k,j}\zeta _1^{\frac{j}{m_k}}\right) \zeta _2^{\frac{k}{m}},\end{aligned}$$

and $\lambda _{\zeta _1}(\lambda _{\zeta _2}(f))=b_{0,0}$, whereas the composition in the other order is not well–defined.

##### Definition 28

( The map ${{\mathscr {L}}_0}$) Let *R* be a real closed field and let $S\subset R\langle \zeta _1, \ldots , \zeta _m\rangle ^n$ be a semialgebraic set bounded over *R*. We denote by

$$\begin{aligned} \mathscr {L}_0 (S):=\lambda _{\zeta _1}\cdots \lambda _{\zeta _m}(S). \end{aligned}$$

##### Remark 29

The set ${{\mathscr {L}}_0}(S)\subset R^n$ is closed. In fact, if *S* is defined by a formula $\psi$ with coefficients in $R\langle \zeta \rangle$, then it follows from [1, Prop. 12.43] that

15 $$\begin{aligned} {{\mathscr {L}}_0}(S)&=\overline{\left\{ (x, t)\in R^{n+1}\,\bigg |\, \left( x\in S|_t\right) \wedge (t>0)\right\} }\cap R^{n} \nonumber \\&=\overline{\textrm{Reali}\bigg (\psi |_{\zeta =t}\wedge (t>0);\mathbb {R}\bigg )}\cap R^{n}, \end{aligned}$$

where we identify $R^{n}$ with $\{u=0\}$. A similar argument holds for $m>1$. Notice, however, that deducing a presentation for $\mathscr {L}_0(S)$ as in Definition 6 is more complicated and requires quantifier elimination, which would bring us back to the problem mentioned in Remark 11.

#### Hausdorff limits

Going back to Example 14, assuming that $A\subset \mathbb {R}^n$ is bounded, then also the corresponding $S\subset \mathbb {R}\langle \zeta \rangle ^n$ is bounded. Moreover, we notice that $\mathscr {L}_0(S)=A.$ Then, the set $\mathcal {U}_r(A)= S|_r$ converges in the Hausdorff metric to $A={{\mathscr {L}}_0}(S)$. To state the analogue result in general, we need to recall some more preliminary notions.

Let *R* be a real closed field. Given a semialgebraic set $S\subset R^n$, the distance from *S* is the function defined by

$$\begin{aligned}\delta _S(x):=\textrm{inf}_{s\in S}\Vert x-s\Vert , \quad x\in R^n.\end{aligned}$$

This is a continuous, semialgebraic function, vanishing on the closure of *S* and positive elsewhere, see [4, Prop. 2.2.8]. Given $S\subset R^n$ and $r\in R$, the *r*–neighbourhood of *S* in $R^n$ is the set defined by

$$\begin{aligned}\mathcal {U}_r(S, R^n):=\left\{ x\in R^n\,\bigg |\, \delta _S(x)\le r\right\} .\end{aligned}$$

Since $\textrm{dist}_S(\cdot )$ is semialgebraic, for every $r>0$ the set $\mathcal {U}_r(S, R^n)$ is also semialgebraic.

##### Definition 30

(Semialgebraic Hausdorff distance)The *Hausdorff distance* between two semialgebraic sets $S_1, S_2\subset R^n$ is defined as

$$\begin{aligned}\textrm{dist}_{H}(S_1, S_2):=\inf \left\{ \epsilon \in R\,\bigg |\, S_1\subseteq \mathcal {U}_\epsilon (S_2),\, S_2\subseteq \mathcal {U}_\epsilon (S_2)\right\} .\end{aligned}$$

Note that, if $R=\mathbb {R}$, this gives the usual Hausdorff distance. However, for general real closed fields *R*, this is not a “distance” in the sense of metric geometry, since the values of this function are elements of *R*. Still, given three closed semialgebraic sets $S_1, S_2, S_3,$ we have

16 $$\begin{aligned} \textrm{dist}_H(S_1, S_3)\le \textrm{dist}_H(S_1, S_2)+\textrm{dist}_H(S_2, S_3).\end{aligned}$$

This is proved exactly as in the classical case $R=\mathbb {R}$.

The next result outlines a useful property related to the map (14).

##### Proposition 31

Let $S_1, S_2\subset R\langle \zeta \rangle _b^n$ be semialgebraic sets. Then

17 $$\begin{aligned} \textrm{dist}_{H}(\lambda _{\zeta }(S_1), \lambda _{\zeta }(S_2))\le \lambda _\zeta \left( \textrm{dist}_H(S_1, S_2)\right) .\end{aligned}$$

##### Proof

We first prove the following fact. If $x, y\in R\langle \zeta \rangle _b^n$, then

18 $$\begin{aligned} \Vert \lambda _{\zeta }(x)-\lambda _\zeta (y)\Vert = \lambda _\zeta \left( \Vert x-y\Vert \right) .\end{aligned}$$

In fact, since $\lambda _\zeta : R\langle \zeta \rangle _b^n\rightarrow R^n$ is a ring homomorphism, writing $u=\sum _{k\ge 0}a_k\zeta ^{\frac{k}{q}}$, with $a_k\in R^n$, it holds

$$\begin{aligned}\Vert \lambda _\zeta (u)\Vert = \Vert a_0\Vert =\lambda _\zeta (\Vert u\Vert ).\end{aligned}$$

Let us now prove (17). Let $\epsilon \in R\langle \zeta \rangle$ such that $\textrm{dist}_H(S_1, S_2)\le \epsilon$. This is equivalent to the following statement: for every $a_1\in S_1$ there exists $a_2(a_1)\in S_2$ such that $\Vert a_1-a_2(a_1)\Vert \le \epsilon$ and for every $b_2\in S_2$ there exists $b_1(b_2)\in S_1$ such that $\Vert b_1(b_2)-b_2\Vert \le \epsilon .$

Then, for every $r_1=\lambda _\zeta (a_1)\in \lambda _\zeta (S_1)$ the element $r_2:=\lambda _\zeta (a_2(a_1))\in \lambda _\zeta (S_2)$ is such that, using (18),

$$\begin{aligned}\Vert r_1-r_2\Vert =\Vert \lambda _\zeta (a_1)-\lambda _\zeta (a_2(a_1))\Vert =\lambda _\zeta (\Vert a_1-a_2\Vert )\le \lambda _\zeta (\epsilon ).\end{aligned}$$

Similarly, for every $s_2\in \lambda _\zeta (S_2)$ there exists and element $s_1\in \lambda _\zeta (S_1)$ such that

$$\begin{aligned}\Vert s_1-s_2\Vert \le \lambda _\zeta (\epsilon ).\end{aligned}$$

This means that $\textrm{dist}_H(\lambda _\zeta (S_1), \lambda _\zeta (S_2))\le \lambda _\zeta (\epsilon )$. $\square$

Recall now the following result from [2].

##### Proposition 32

([2, Prop. 2.7]) Let $S=\textrm{Reali}(\psi ; \mathbb {R}\langle \zeta \rangle )\subset \mathbb {R}\langle \zeta \rangle ^n$ be a bounded semialgebraic set. Then, in the usual Hausdorff metric,

$$\begin{aligned} \lim _{r\rightarrow 0} S|_r={{\mathscr {L}}_0}(S). \end{aligned}$$

##### Remark 33

The previous result is stated in [2, Prop. 2.7] under the assumption that the formula $\psi$ has coefficients in $\mathbb {R}[\zeta ]$. In fact the proof in the general case goes exactly in the same way, provided that *r* is sufficiently small so that the evaluation is well–defined as explained in Definition 16.

##### Remark 34

Even if $S|_0$ is well–defined, in general, this may be far, in the Hausdorff metric, from $\mathscr {L}_0(S)$. For example let $\alpha =\left( x^2-\zeta ^2<0\right)$ and $A=\textrm{Reali}(\alpha ; R\langle \zeta \rangle )$. Then $A|_0=\emptyset$ and $\mathscr {L}_0(A)=\{0\},$ so that $A|_0\subsetneq \mathscr {L}_0(A)$. On the other hand, if $\beta =(\zeta x=0)$, and $B=\textrm{Reali}(\beta ; R\langle \zeta \rangle )$, then $B|_0=R$ and $\mathscr {L}_0(B)=\{0\}$, so that $B|_0\supsetneq \mathscr {L}_0(B).$

We extend Proposition 32 to any real closed field using the transfer principle.

##### Proposition 35

Let *F* be a real closed extension of $\mathbb {R}$. Let $S=\textrm{Reali}(\psi ; F\langle \zeta \rangle )\subset F\langle \zeta \rangle ^n$ be a bounded semialgebraic set. Then,

$$\begin{aligned} \forall \epsilon>0 \quad \exists \delta >0\quad \forall 0< t<\delta \quad \textrm{dist}_H(\textrm{Reali}(\psi |_{\zeta = t}; F), \lambda _\zeta (S))<\epsilon . \end{aligned}$$

(Here all the variables $\epsilon , \delta ,t$ are in *F*, and $\textrm{dist}_H$ denotes the semialgebraic Hausdorff distance.)

##### Proof

Given a semialgebraic set *S* as in the statement of Proposition 32, we can rephrase the content of its conclusion by saying that,

$$\begin{aligned} \forall \epsilon>0 \quad \exists \delta >0\quad \forall 0<t<\delta \quad \textrm{dist}_H(\textrm{Reali}(\psi |_{\zeta =t};\mathbb {R}), \lambda _\zeta (S))<\epsilon . \end{aligned}$$

All variables in this formula are in $\mathbb {R}$ and $\textrm{dist}_H$ is the usual Hausdorff distance, which is written as a first order formula with coefficients in $\mathbb {R}$. Therefore, the same conclusion holds if $\mathbb {R}$ is replaced by a real closed extension *F* of it, by [4, Prop. 5.2.3]. $\square$

We now generalize Proposition 35 to the case of multiple infinitesimals.

##### Theorem 36

Let *R* be a real closed extension of $\mathbb {R}$. Let $S=\textrm{Reali}(\psi ;R\langle \zeta _1, \ldots , \zeta _m\rangle )\subset R\langle \zeta _1, \ldots , \zeta _m\rangle ^n$ be a semialgebraic set, bounded over *R*. Then, for every $1\le k\le m$ it holds

19 $$\begin{aligned} \text {``}\lim _{t_k\rightarrow 0}\cdots \lim _{t_m\rightarrow 0}S|_{t_k, \ldots , t_m}=\lambda _{\zeta _k}\cdots \lambda _{\zeta _m}(S)\text {''}, \end{aligned}$$

where (19) means: for all $\epsilon \in R\langle \zeta _1, \ldots , \zeta _{k-1}\rangle$, $\epsilon >0$, for $0<t_k\ll \cdots \ll t_m\ll 1$ it holds

$$\begin{aligned} \textrm{dist}_H\Big (S|_{t_k, \ldots , t_m}, \lambda _{\zeta _k}\cdots \lambda _{\zeta _m}(S)\Big )< \epsilon , \end{aligned}$$

where $\textrm{dist}_H$ is the semialgebraic Hausdorff distance (see Definition 30).

##### Proof

When $k=m$, the statement is given by Proposition 35 applied to the case $F=R\langle \zeta _1, \ldots , \zeta _{m-1}\rangle$, so that $F\langle \zeta \rangle \simeq R\langle \zeta _1, \ldots , \zeta _{m-1}, \zeta \rangle$, where $\zeta =\zeta _{m}$.

Assume that the statement is true for $k=j\le m$. We prove it for $k=j-1.$ By our working assumption, for every $\epsilon \in R\langle \zeta _1, \ldots , \zeta _{j-1}\rangle$, $\epsilon >0$, for $0<t_j\ll t_{j+1}\ll \dots \ll t_m \ll 1$ the following property holds:

$$\begin{aligned} \textrm{dist}_{H}\left( S|_{t_j, \ldots , t_m}, \lambda _{\zeta _j}\cdots \lambda _{\zeta _m}(S)\right) < \frac{\epsilon }{2}. \end{aligned}$$

Recall that here $t_i \in R\langle \zeta _1, \ldots , \zeta _{i-1}\rangle$ for every $j\le i\le m$. A fortiori we can take $\epsilon \in R\langle \zeta _1, \ldots , \zeta _{j-2}\rangle$.

Denote by $A:=S|_{t_j, \ldots , t_m}\subset R\langle \zeta _1, \ldots , \zeta _{j-1}\rangle ^n$ and by $B:=\lambda _{\zeta _j}\cdots \lambda _{\zeta _m}(S)\subset R\langle \zeta _1, \ldots , \zeta _{j-1}\rangle ^n$, which are both bounded over *R*. Given $\epsilon \in R\langle \zeta _1, \ldots , \zeta _{j-2}\rangle$, $\epsilon >0$ let $\delta _{j-1}\in R\langle \zeta _1, \ldots , \zeta _{j-2}\rangle$, $\delta _{j-1}>0$ be given by Proposition 35 such that for all $t_{j-1} \in R\langle \zeta _1,\dots ,\zeta _{j-2}\rangle$ with $0<t_{j-1}<\delta _{j-1}$ the following property holds:

20 $$\begin{aligned} \textrm{dist}_{H}\left( A|_{t_{j-1}}, \lambda _{\zeta _{j-1}}(A)\right) < \frac{\epsilon }{2}. \end{aligned}$$

Then, for $0<t_{j-1}\ll t_j \ll \dots \ll t_m \ll 1$ it holds

$$\begin{aligned} \textrm{dist}_H(S|_{t_{j-1}, \ldots , t_m}, \lambda _{\zeta _{j-1}}\cdots \lambda _{\zeta _m}(S))&{\mathop {\le }\limits ^{(16)}} \textrm{dist}_H(A|_{t_{j-1}}, \lambda _{\zeta _{j-1}}(A))\\&\quad +\textrm{dist}_H(\lambda _{\zeta _{j-1}}(A), \lambda _{\zeta _{j-1}}(B)),\\&{\mathop {<}\limits ^{(20)}} \frac{\epsilon }{2}+\lambda _{\zeta _{j-1}}\left( \textrm{dist}_H(A,B)\right) ,\\&{\mathop {\le }\limits ^{(17)}}\frac{\epsilon }{2}+\lambda _{\zeta _{j-1}}\left( \frac{\epsilon }{2}\right) = \epsilon , \end{aligned}$$

where in the last inequality we used the fact that $\epsilon$ was chosen in $R\langle \zeta _1, \ldots , \zeta _{j-2}\rangle$. $\square$

### Hausdorff approximations of closed and bounded sets

Recall that every closed (in the Euclidean topology) semialgebraic set $S\subseteq R^n$ can be written as a finite union of closed basic semialgebraic sets ([4, Thm. 2.7.2]):

21 $$\begin{aligned} S=\bigcup _{i=1}^{a}\bigcap _{j=1}^{b_i}\{x\in R^n \mid p_{ij}(x)\le 0\}. \end{aligned}$$

In general, for a closed semialgebraic set *S* with diagram $D(S)=(n, c, d)$, passing from a representation of the form (4) to one of the form (21), we cannot control the number of unions and intersections in (21) as a function of *c*, nor the degrees of the polynomials in (21) as a function of *d*. (On the other hand, in passing from (21) to (4) the process is controlled.)

However, here we use the tools from the previous section to show that, given a closed and bounded semialgebraic set with a representation as in (4), we can *approximate* it with a closed and bounded semialgebraic set described as in (21) in a controlled way (Proposition 38).

We start with the following elementary lemma.

#### Lemma 37

Let $C \subset \mathbb {R}^n$ be of the form

$$\begin{aligned} C=\left( \bigcap _{j\in J^=}\left\{ x\in \mathbb {R}^n\,\bigg |\,p_{j}(x)=0\right\} \right) \cap \left( \bigcap _{j\in J^{<}}\left\{ x\in \mathbb {R}^n\,\bigg |\,q_{j}(x)<0\right\} \right) , \end{aligned}$$

where the $p_j$ and the $q_j$ are polynomials and $J^=, J^<$ are finite sets. Then, denoting by (*x*, *u*) points in $\mathbb {R}^n \times \mathbb {R}$, and identifying $\{u=0\}$ with $\mathbb {R}^n$, the closure of *C* can be described as:

22 $$\begin{aligned} \overline{C}= &   \overline{\left\{ (x, u) \, \bigg | \bigg ( p_j (x) = 0,\, \forall j\in J^=\bigg )\wedge \bigg (q_j (x) + u \le 0, \, \forall j\in J^<\bigg )\wedge \bigg ( u>0\bigg ) \right\} }\nonumber \\  &   \,\, \cap \{u=0\}. \end{aligned}$$

#### Proof

We prove the two inclusions separately. To prove the inclusion of the set on the left of (22) into the one on the right, by the monotonicity of the closure operation, it is enough to show that for any $z \in C$ there exists a sequence $\{ (z_k,u_k)\}_{k\ge 1}$ converging to (*z*, 0) satisfying for every $k\ge 1$

$$\begin{aligned} \bigg ( p_j (z_k) = 0,\, \forall j\in J^=\bigg )\wedge \bigg (q_j (z_k) + u_k \le 0, \, \forall j\in J^<\bigg )\wedge \bigg ( u_k>0\bigg ). \end{aligned}$$

Since $z \in C$, we have

$$\begin{aligned} \bigg ( p_j (z) = 0,\, \forall j\in J^=\bigg ) \wedge \bigg (q_j (z) + U \le 0, \, \forall j\in J^<\bigg ), \end{aligned}$$

with $U:= - \max \{q_j (z) \,|\, j\in J^< \} > 0$. Hence, setting $u_k:= \frac{U}{k} >0$ and $z_k:= z$, we observe that the sequence $(z_k, u_k)$ satisfies all the above conditions and converges to (*z*, 0). To prove the other inclusion, we take

$$\begin{aligned} ( z, 0) \in \overline{\left\{ (x, u)\in \mathbb {R}^n \times \mathbb {R}\, \bigg | \bigg ( p_j (x) = 0,\, \forall j\in J^=\bigg )\wedge \bigg (q_j (x) + u \le 0, \, \forall j\in J^<\bigg )\wedge \bigg ( u>0\bigg ) \right\} }. \end{aligned}$$

Hence, there exists a sequence $(z_k, u_k)$ converging to (*z*, 0) such that for any *k*

$$\begin{aligned} \bigg ( p_j (z_k) = 0,\, \forall j\in J^=\bigg )\wedge \bigg (q_j (z_k) + u_k \le 0, \, \forall j\in J^<\bigg )\wedge \bigg ( u_k>0\bigg ). \end{aligned}$$

In particular $z_k \in C$ for all *k*, hence $z \in \overline{C}$. $\square$

#### Proposition 38

For every $\epsilon >0$ and for every closed and bounded semialgebraic set $S\subset \mathbb {R}^n$ with diagram $D(S)=(n, c, d)$, there exists a closed semialgebraic set $S'\subset \mathbb {R}^n$ satisfying

$$\begin{aligned}\textrm{dist}_{H}(S, S')\le \epsilon \end{aligned}$$

and such that

$$\begin{aligned}{S'} = \bigcup _{i=1}^a \textrm{Bas}(\mathcal {P}_{i}, \mathcal {Q}_i;\mathbb {R}),\end{aligned}$$

with the property that, for every $i=1, \ldots , a$ we have $\#(\mathcal {P}_i\cup \mathcal {Q}_i)\le b$, with $ab\le c$, and with each polynomial in $\mathcal {P}_i\cup \mathcal {Q}_i$ of degree bounded by *d*.

#### Proof

Let us write

$$\begin{aligned}S=\bigcup _{i=1}^{a} \bigcap _{j=1}^{b_i}\left\{ x\in \mathbb {R}^n\,\bigg |\,\textrm{sign}(p_{ij}(x))=\sigma _{ij}\right\} , \end{aligned}$$

with $c=a\max _i\{b_i\}$. Let us examine the sets

$$\begin{aligned}C_i:=\bigcap _{j=1}^{b_i}\left\{ x\in \mathbb {R}^n\,\bigg |\,\textrm{sign}(p_{ij}(x))=\sigma _{ij}\right\} .\end{aligned}$$

By possibly relabelling the $p_{ij}$ and multiplying them by $\pm 1$, we can write each $C_i$ as

$$\begin{aligned}C_i=\left( \bigcap _{j\in J^=_{i}}\left\{ x\in \mathbb {R}^n\,\bigg |\,p_{ij}(x)=0\right\} \right) \cap \left( \bigcap _{j\in J_{i}^{<}}\left\{ x\in \mathbb {R}^n\,\bigg |\,p_{ij}(x)<0\right\} \right) ,\end{aligned}$$

where $\#\left( J_i^=\cup J_i^<\right) = b_i.$ For each $i=1, \ldots , a$ and for every $i\in J_{i}^<$ we define the polynomial $\widetilde{p}_{ij}\in \mathbb {R}\langle \zeta \rangle [x_1, \ldots , x_n]$ as follows:

$$\begin{aligned} \widetilde{p}_{ij}(x):=p_{ij}(x)+\zeta . \end{aligned}$$

For any $i= 1, \dots , a$ we consider the semialgebraic set $\widetilde{S}_i \subset \mathbb {R}\langle \zeta \rangle ^n$ defined by the formula:

$$\begin{aligned} \widetilde{\psi }_i:= \left( \bigwedge _{j\in J_i^=}p_{ij}(x)=0\right) \wedge \left( \bigwedge _{j\in J_{i}^<}\widetilde{p}_{ij}(x)\le 0\right) . \end{aligned}$$

We claim that, if $S\subseteq B_{\mathbb {R}^n}(\rho )$ for $\rho >0$, then $\widetilde{S}_i \subseteq B_{\mathbb {R}\langle \zeta \rangle ^n}(\rho )$. In fact, let $g:(0, \delta )\rightarrow \mathbb {R}$ be a representative for an element of $\widetilde{S}_i$. Then, for every $\zeta \in (0, \delta )$ we have $p_{ij}(g(\zeta ))=0$ for $j\in J_i^=$ and $p_{ij}(g(\zeta ))+\zeta \le 0$ for $j\in J_i^{<}$. Since $\zeta <\delta$, the inequality $p_{ij}(g(\zeta ))+\zeta \le 0$ implies $p_{ij}(g(\zeta ))<0$, i.e. $g(\zeta )\in C_i\subseteq B_{\mathbb {R}^n}(\rho )$ for every $\zeta \in (0, \delta )$. Therefore $g\in B_{\mathbb {R}\langle \zeta \rangle ^n}(\rho )$.

In particular $\widetilde{S}_i$ is bounded and, by Proposition 32, for any $\epsilon >0$ we get $r >0$ such that

23 $$\begin{aligned} \textrm{dist}_{H} \left( { {\mathscr {L}}_0}(\widetilde{S}_i), \widetilde{S}_i|_{r}) \right) \le \epsilon . \end{aligned}$$

We have by (15) and Lemma 37

$$\begin{aligned} {\mathscr {L}}_0 (\widetilde{S}_i) = \overline{ \left\{ (x, u)\in \mathbb {R}^{n+1}\, \bigg |\, \left( x\in \widetilde{S}_i|_{u} \right) \wedge (u>0) \right\} } \cap \mathbb {R}^{n} = \overline{C_i}. \end{aligned}$$

Observe that $\widetilde{S}_i|_{r} = \textrm{Bas}(\mathcal {P}_{i}, \mathcal {Q}_i;\mathbb {R})$, where

$$\begin{aligned} \mathcal {P}_i=\left\{ p_{ij}\,\big |\, j\in {J_i^=}\right\} \quad \text {and}\quad \mathcal {Q}_i = \left\{ p_{ij}+r \,\big |\, j\in J_{i}^< \right\} . \end{aligned}$$

Note that $\#(\mathcal {P}_i\cup \mathcal {Q}_i)\le b:=\max _i b_i$ so that $ab\le c$, and each polynomial in $\mathcal {P}_i\cup \mathcal {Q}_i$ has degree bounded by *d*. We now define

$$\begin{aligned}S': = \bigcup _{i=1}^a C_i',\qquad \text {with} \qquad C_i':=\textrm{Bas}(\mathcal {P}_{i}, \mathcal {Q}_i;\mathbb {R}). \end{aligned}$$

Since *S* is closed, we have $S=\bigcup _{i=1}^a \overline{C_i}$. From (23) we get directly

$$\begin{aligned} \textrm{dist}_{H} ( S, S')\le \max _i \textrm{dist}_H\left( \overline{C_i},C_i'\right) \le \epsilon , \end{aligned}$$

concluding the proof. $\square$

## Quantitative approximate definable choices

### Preliminary constructions

Following a construction from [3], given a closed basic semialgebraic set

$$\begin{aligned} S=\textrm{Bas}(\mathcal {P}, \mathcal {Q};\mathbb {R})\subset \mathbb {R}^{n} \end{aligned}$$

we construct a closed basic semialgebraic set $\widetilde{S} \subset R^{n}$, where *R* is a field of algebraic Puiseux series with coefficients in $\mathbb {R}$, such that $\mathscr {L}_0(\widetilde{S})=S$ and for which a definable choice over a given projection can be made quantitatively.

#### Construction 39

The following construction is taken from [3]. Set

$$\begin{aligned} R= \mathbb {R}\langle \zeta _1, \zeta _2, \zeta _3\rangle . \end{aligned}$$

For a given closed basic and bounded semialgebraic set

$$\begin{aligned} S=\textrm{Bas}(\mathcal {P}, \mathcal {Q};\mathbb {R})\subset \mathbb {R}^{n},\end{aligned}$$

and for every $1\le k\le n$ the construction provides new semialgebraic sets

$$\begin{aligned} \widetilde{S}^k\subseteq \widetilde{S}\subset R^{n}. \end{aligned}$$

Assume that the polynomials in $\mathcal {P}, \mathcal {Q}$ have degree bounded by *d*. First, using a perturbation argument, one constructs new families of polynomials $\widetilde{\mathcal {P}}, \widetilde{\mathcal {Q}}\subset R[x_1, \ldots , x_{n}]$ with degrees bounded by $2d+2$. The cardinality of $\widetilde{\mathcal {Q}}$ equals the one of $\mathcal {Q}$, whereas (for our purposes) the cardinality of $\widetilde{\mathcal {P}}$ can be assumed to be 1 (by taking the sum of the squares of its elements). In this way we get the set

$$\begin{aligned} \widetilde{S}:=\textrm{Bas}(\widetilde{\mathcal {P}}, \widetilde{\mathcal {Q}}; R). \end{aligned}$$

Then, one constructs $\widetilde{S}^k$. First, set

$$\begin{aligned}g(x):=1+\sum _{j=1}^{n}j x_j^{2d+2},\end{aligned}$$

and for every family $\mathcal {F}=\{f_1,\ldots , f_s\}\subset R[x_1, \ldots , x_{n}]$ define the algebraic set:

24 $$\begin{aligned} \textrm{Crit}_k(\mathcal {F}; R):=\{x\in R^{n}\mid f_1(x)=\cdots =f_s(x)=0,\, \textrm{rank}(\widetilde{J}(x))\le k\}, \end{aligned}$$

where $\widetilde{J}(x)$ is the matrix of size $(n-k)\times (s+1)$ whose columns are the partial derivatives of $f_1, \ldots , f_s, g$ with respect to $x_{k+1}, \ldots , x_{n}$. The set $\widetilde{S}^k$ is then defined by

25 $$\begin{aligned} \widetilde{S}^k:=\bigcup _{\widetilde{\mathcal {Q}}'\subset \widetilde{\mathcal {Q}}}\textrm{Crit}_k(\widetilde{\mathcal {P}}\cup \widetilde{\mathcal {Q}}'; R)\cap \widetilde{S},\end{aligned}$$

where the union runs over all subsets $\widetilde{\mathcal {Q}}'\subset \widetilde{\mathcal {Q}} \subset R[x_1,\dots ,x_n]$.

#### Remark 40

(On boundedness) Even though this is not explicitly stated in [3] (but indeed used in their arguments), by inspecting the form of $\widetilde{\mathcal {P}}$ and $\widetilde{\mathcal {Q}}$ provided there, one can see that, if $S\subset \mathbb {R}^{n}$ is bounded, then $\widetilde{S}^\ell \subseteq \widetilde{S}\subset R^{n}$ are bounded over $\mathbb {R}$.

The next result gives a control on the diagram of $\widetilde{S}^k$.

#### Lemma 41

For every $a_1\in \mathbb {N}$ there exist $a_2, a_3\in \mathbb {N}$ such that, for a closed basic semialgebraic set $S=\textrm{Bas}(\mathcal {P}, \mathcal {Q};\mathbb {R})\subset \mathbb {R}^{n}$, with $\#\mathcal {P}, \#\mathcal {Q}\le a_1$ and with every polynomial in $\mathcal {P}\cup \mathcal {Q}$ of degree bounded by *d*, and any $1\le k \le n$, it holds

$$\begin{aligned} D(\widetilde{S}^{k})=(n, a_2, a_3 d), \end{aligned}$$

where $\widetilde{S}^{k} \subset R^{n}$ is the set associated with *S* by Construction 39.

#### Proof

Let $\widetilde{\mathcal {P}}, \widetilde{\mathcal {Q}}\subset R[x_1, \ldots , x_{n}]$ be the family of polynomials of Construction 39 such that $\widetilde{S}=\textrm{Bas}(\widetilde{\mathcal {P}}, \widetilde{\mathcal {Q}};R)$. These polynomials have degree bounded by $2d+2$.

Then, for every subset $\widetilde{\mathcal {Q}}'\subset \widetilde{\mathcal {Q}}$ (there are only finitely many such subsets, say at most $a_4=a_4(a_1)$) one considers the family $\mathcal {F}=\widetilde{\mathcal {P}}\cup \widetilde{\mathcal {Q}}'=\{f_1, \ldots , f_s\}$ (here $s\le a_4+1$) and defines the algebraic set $\textrm{Crit}_{k}(\mathcal {F})$ as in (24). Since *R* is real closed, this algebraic set is defined by a single polynomial equation $P_{\widehat{\mathcal {Q}}'}(x)=0$, where

$$\begin{aligned} P_{\widehat{\mathcal {Q}}'}=\sum _{i=1}^s f_i^2+\sum m_{ij}^2. \end{aligned}$$

Here, the second sum runs over all the $(k+1)\times (k+1)$ minors of $\widetilde{J}(x)$. Remember that, $\widetilde{J}(x)$ is a $(n-k)\times (s+1)$ matrix, hence if $k> s$ there are no such minors, so we can assume that $k\le s$. Therefore the polynomial $P_{\widehat{\mathcal {Q}}'}$ has degree

$$\begin{aligned} \deg \left( P_{\widehat{\mathcal {Q}}'}\right) \le (k+1)(4d+4) \le (s+1)(4d+4) \le a_5 d, \end{aligned}$$

where $$a_5=a_5(a_1)$$.

The set $\widetilde{S}^\ell$ from Construction 39 can therefore be written as:

26 $$\begin{aligned} \widetilde{S}^\ell&= \bigcup _{\widetilde{\mathcal {Q}}'\subset \widetilde{\mathcal {Q}}}\textrm{Crit}_{k}(\widetilde{\mathcal {P}}\cup \widetilde{\mathcal {Q}}';R)\cap \widetilde{S}=\widetilde{S}\cap \bigcup _{\widehat{\mathcal {Q}}'\subset \widehat{\mathcal {Q}}}Z(P_{\widehat{\mathcal {Q}}'};R)=\widetilde{S}\cap Z(\widetilde{F};R) \nonumber \\&= Z(\widetilde{\mathcal {P}};R) \bigcap _{\tilde{q}\in \widetilde{\mathcal {Q}}} \{\tilde{q} \le 0\} \cap Z(\widetilde{ F};R), \end{aligned}$$

where $\widetilde{\mathcal {P}},\widetilde{\mathcal {Q}}$ are the families defining the basic set $\widetilde{S}$, and

$$\begin{aligned} \widetilde{F}:=\prod _{\widetilde{\mathcal {Q}}'\subset \widetilde{\mathcal {Q}}}P_{\widetilde{\mathcal {Q}}'}, \end{aligned}$$

which is a polynomial of degree bounded by $a_4a_5 d=a_3 d$, with $a_3$ depending only on $a_1$. To obtain a presentation as in (4) note that if $\{p_1,\dots ,p_L\}$ is a family of polynomials, then

27 $$\begin{aligned} \bigcap _{i =1}^L \{ p_i \le 0\} = \bigcap _{i = 1}^L \Big ( \{ p_i < 0\} \cup \{p_i = 0\} \Big ) = \bigcup _{\sigma } \bigcap _{i=1}^L \{\textrm{sign}(p_i) = \sigma _i\}, \end{aligned}$$

where $\sigma$ runs over all possible choices in $\{0,-1\}^{L}$. We can apply identity (27) to (26). Since $\#\widetilde{\mathcal {P}}, \#\widetilde{\mathcal {Q}}\le a_1$, we get that $\widetilde{S}^\ell$ admits a representation as in (4) with $a=2^L$, $b_i = L$, for $L=2a_1+1$. In other words $D(\widetilde{S}^\ell )=(n,a_2,a_3 d)$, for $a_2 = (2a_1+1)2^{2a_1+1}$. $\square$

The sets from Construction 39 enjoy the following properties. For $1\le k\le n$ let

$$\begin{aligned} \pi _k:R^{n}\rightarrow R^k \end{aligned}$$

be the projection onto the last *k* coordinates. Since $\mathbb {R}\subset R =\mathbb {R}\langle \zeta _1,\zeta _2,\zeta _3\rangle$, the same symbol $\pi _k:\mathbb {R}^{n}\rightarrow \mathbb {R}^k$ is used to denote the restriction to $\mathbb {R}^n$, without risk of confusion.

#### Proposition 42

Let $S=\textrm{Bas}(\mathcal {P}, \mathcal {Q};\mathbb {R})\subset \mathbb {R}^{n}$ be a closed basic and bounded semialgebraic set such that, for some $1\le k\le n$ it holds

28 $$\begin{aligned} \forall y\in \mathbb {R}^k\quad \#\left( Z(\mathcal {\mathcal {P}};\mathbb {R})\cap \pi _{k}^{-1}(y)\right) <\infty , \end{aligned}$$

i.e. $Z(\mathcal {\mathcal {P}};\mathbb {R})$ is strongly of dimension $\le k$. Then for any $\ell < k$, the sets $\widetilde{S}^\ell \subseteq \widetilde{S}\subset \mathbb {R}\langle \zeta _1,\zeta _2,\zeta _3\rangle ^n$ from Construction 39 are closed and bounded over $\mathbb {R}$. Moreover they satisfy the following properties. For every $w\in \mathbb {R}\langle \zeta _1,\zeta _2,\zeta _3\rangle ^\ell$

(i) the set $\widetilde{S}^\ell \cap \pi _\ell ^{-1}(w)$ is finite, i.e. $\widetilde{S}^\ell$ is strongly of dimension $\le \ell$;
(ii) if $\widetilde{S}\cap \pi _{\ell } ^{-1}(w)\ne \emptyset$, the set $\widetilde{S}^\ell \cap \pi _\ell ^{-1}(w)$ intersects every connected component of $\widetilde{S}\cap \pi _\ell ^{-1}(w)$;
(iii) it holds ${{\mathscr {L}}_0}(\widetilde{S}) = S$.

Furthermore, for every $\epsilon \in \mathbb {R}$, $\epsilon >0$, for $0<t_1\ll \dots \ll t_m \ll 1$ the evaluation $S|_{t_1,\dots ,t_m}$ is defined and the following properties hold:

(iv) $$\displaystyle \textrm{dist}_H(\widetilde{S}|_{t_1,\dots ,t_m},S)<\epsilon $$;
(v) $$\displaystyle \textrm{dist}_H\left( \pi _\ell (\widetilde{S}|_{t_1,\dots ,t_m}),\pi _\ell (S)\right) <\epsilon $$.

#### Proof

The fact that $\widetilde{S}^\ell \subseteq \widetilde{S}\subset \mathbb {R}\langle \zeta _1,\zeta _2,\zeta _3\rangle ^n$ are closed, and bounded follows directly by their construction. Items (i) to (iii) are proved in [3, Prop. 5.5 and Prop. 5.17], while Item (iv) follows from Item (iii) and Proposition 36. To conclude, observe that $\pi _\ell : \mathbb {R}^n\rightarrow \mathbb {R}^\ell$ is 1-Lipschitz with respect to $\textrm{dist}_H$, therefore Item (v) follows from Item (iv). $\square$

### Approximate definable choice: the case of a projection

The purpose of this section is to prove Theorem A. We begin with the following preliminary version of the latter, for the case of closed basic sets.

#### Proposition 43

Let $\pi :\mathbb {R}^{n}\rightarrow \mathbb {R}^\ell$, with $1\le \ell \le n$ be the projection onto the last $\ell$ coordinates. For every $a_1\in \mathbb {N}$ there exist $a_2, a_3\in \mathbb {N}$ such that the following holds. For every closed basic and bounded semialgebraic set

$$\begin{aligned} S=\textrm{Bas}(\mathcal {P}, \mathcal {Q};\mathbb {R}) \subset \mathbb {R}^{n}, \end{aligned}$$

with $\#\mathcal {P}, \#\mathcal {Q}\le a_1$ and with every polynomial in $\mathcal {P}\cup \mathcal {Q}$ of degree bounded by *d*, and for all $\epsilon >0$ there exists a semialgebraic set $A_\epsilon \subset \mathbb {R}^{n}$ satisfying the following properties:

(i) $$\dim (A_\epsilon )\le \ell $$;
(ii) $$A_\epsilon \subseteq \mathcal {U}_\epsilon (S)$$;
(iii) $$\textrm{dist}_{H}(\pi (A_\epsilon ), \pi (S))\le \epsilon $$;
(iv) $$D(A_\epsilon )=(n, a_2, a_3 d)$$.

#### Proof

If $\dim (S)\le \ell$ and one can simply take $A_\epsilon =S$.

Assume then that $k=\dim (S)>\ell$. Let $\widetilde{S}^\ell \subseteq \widetilde{S} \subset R^{n}$, where $R = \mathbb {R}\langle \zeta _1,\zeta _2,\zeta _3\rangle$ be the set defined by Construction 39. The strategy is to define

29 $$\begin{aligned} A_\epsilon :=\widetilde{S}^\ell |_{t_1,t_2,t_3} \end{aligned}$$

for an appropriate choice of “sufficiently small” $(t_1,t_2,t_3)$ and to use Proposition 42. However, in order to use it we need first to ensure that the condition (28) is satisfied. In order to do it, using Proposition 13, we can first perform a small linear change of variables $L:\mathbb {R}^{n}\rightarrow \mathbb {R}^{n}$ (i.e. we choose *L* sufficiently close to the identity) in the defining polynomials and get new families $\mathcal {P}', \mathcal {Q}'$ with the property that: $\#\mathcal {P}'=\#\mathcal {P}$, $\#\mathcal {Q}'=\#\mathcal {Q}$; the degrees of all elements of $\mathcal {P}', \mathcal {Q}'$ are bounded by *d*; the Hausdorff distance between $\textrm{Bas}(\mathcal {P}, \mathcal {Q};\mathbb {R})$ and $\textrm{Bas}(\mathcal {P}', \mathcal {Q}';\mathbb {R}){ = L (\textrm{Bas}(\mathcal {P}, \mathcal {Q};\mathbb {R}))}$ is arbitrarily small; and condition (28) is satisfied.

It is elementary to verify that a set $A_\epsilon '\subset \mathbb {R}^{n}$ satisfying the properties of the statement for the semialgebraic set $\textrm{Bas}(\mathcal {P}',\mathcal {Q}';\mathbb {R})$ will also satisfy the same properties for the original set $\textrm{Bas}(\mathcal {P}, \mathcal {Q};\mathbb {R})$, up to adjusting $\epsilon$. Therefore, without loss of generality, we assume that (28) is verified for the set *S*.

Set $A_\epsilon$ as in (29). We show how to chose $(t_1,t_2,t_3)$ so that the desired properties hold.

(i) By Item (i) of Proposition 42, the set $\widetilde{S}^\ell$ is strongly of dimension $\le \ell$. By Lemma 22 for $0<t_1 \ll t_2 \ll t_3 \ll 1$ also $\widetilde{S}^\ell |_{t_1,t_2,t_3}$ is strongly of dimension $\le \ell$.
(ii) By Item (iv) of Proposition 42, for $0<t_1 \ll t_2 \ll t_3\ll 1$ we have
  $$\begin{aligned} \textrm{dist}_H(\widetilde{S}|_{t_1,t_2,t_3},S)<\epsilon . \end{aligned}$$
  Since $\widetilde{S}^\ell \subseteq \widetilde{S}$ (in fact the formula defining $\widetilde{S}^\ell$ contains the formula defining $\widetilde{S}$ as a conjunction, by definition (25)), for such $(t_1,t_2,t_3)$ we also have $\widetilde{S}^{\ell }|_{t_1,t_2,t_3} \subseteq \mathcal {U}_\epsilon (S)$.
(iii) By Item v of Proposition 42, for $0<t_1 \ll t_2 \ll t_3\ll 1$ we have
  $$\begin{aligned} \textrm{dist}_H(\pi (\widetilde{S}|_{t_1,t_2,t_3}),\pi (S))<\epsilon . \end{aligned}$$
(iv) By Lemmas 20 and 41, for $0<t_1 \ll t_2 \ll t_3\ll 1$ we have
  $$\begin{aligned} D(\widetilde{S}^\ell |_{t_1,t_2,t_3})=D(\widetilde{S}^\ell )=( n, a_2, a_3 d), \end{aligned}$$
  where $a_2,a_3 \in \mathbb {N}$ depend only on $a_1$.

Since we are requiring only a finite number of properties, given $\epsilon >0$, the quantifier $0<t_1 \ll t_2 \ll t_3\ll 1$ can be commonly chosen (see Definition 15) so that the set (29) satisfies simultaneously Items (i) to (iv). $\square$

Extending the previous result to general (non-basic) semialgebraic sets, we obtain the following statement, that corresponds to Theorem A.

#### Theorem 44

For every $c \in \mathbb {N}$ there exist $\kappa \in \mathbb {N}$ such that the following holds. Let $n,\ell ,d\in \ell$, with $1\le \ell \le n$. Let $\pi :\mathbb {R}^{n}\rightarrow \mathbb {R}^\ell$ be the projection onto the last $\ell$ coordinates and let $S\subset \mathbb {R}^{n}$ be a bounded closed semialgebraic set with

$$\begin{aligned} D(S)=(n, c, d). \end{aligned}$$

Then, for every $\epsilon >0$ there exists a closed semialgebraic set $A_\epsilon \subset \mathbb {R}^{n}$ such that:

(i) $$\dim (A_\epsilon )\le \ell $$;
(ii) $$A_\epsilon \subseteq \mathcal {U}_\epsilon (S)$$;
(iii) $$\textrm{dist}_{H}(\pi (A_\epsilon ), \pi (S))\le \epsilon $$;
(iv) $$D(A_\epsilon )=(n, \kappa , \kappa d)$$.

#### Proof

Applying Proposition 38 we find a closed semialgebraic set $S'\subset \mathbb {R}^{n}$ satisfying

30 $$\begin{aligned} \textrm{dist}_{H}(S, S')\le \frac{\epsilon }{2}, \end{aligned}$$

(in particular, $S'$ is also bounded) and such that

$$\begin{aligned} S'=\bigcup _{i=1}^a \textrm{Bas}(\mathcal {P}_{i}, \mathcal {Q}_i;\mathbb {R}), \end{aligned}$$

with the property that, for every $i=1, \ldots , a$ we have $\#(\mathcal {P}_i\cup \mathcal {Q}_i)\le b$, with $ab\le c$, and with each polynomial in $\mathcal {P}_i\cup \mathcal {Q}_i$ of degree bounded by *d*. For every $i=1, \ldots , a$, denote by $S_i':=\textrm{Bas}(\mathcal {P}_{i}, \mathcal {Q}_i;\mathbb {R})$ and apply Proposition 43 to each $S_i'$ to get sets $A_{i, \epsilon }$ satisfying the conclusions of Proposition 43 (with $\varepsilon /2$ in place of $\varepsilon$). Define

31 $$\begin{aligned} A_\epsilon :=\bigcup _{i=1}^a A_{i,\epsilon }. \end{aligned}$$

(i) Since for every $i=1, \ldots , a$ we have $\dim (A_{i, \epsilon })\le \ell$, Item (i) follows.
(ii) As for Item (ii), this follows from the fact that $A_\epsilon \subseteq \mathcal {U}_\frac{\epsilon }{2}(S')$ and from (30).
(iii) Similarly, for Item (iii), we have
  $$\begin{aligned} \textrm{dist}_H(\pi (A_\epsilon ), \pi (S))\le \textrm{dist}_H(\pi (A_\epsilon ), \pi (S'))+\textrm{dist}_H(\pi (S'), \pi (S))\le \frac{\epsilon }{2}+\frac{\epsilon }{2} \le \epsilon . \end{aligned}$$
(iv) By Item (iv) from Proposition 43, $D(A_{i, \epsilon })= (n, a_2(b), a_3(b)d)$. Therefore $D(A_\epsilon )= (n, \kappa ', \kappa ' d)$ for some $\kappa '=\kappa '(a_2(b), a_3(b), a)\le \kappa (c)$. This proves Item (iv).

The set $A_\epsilon$ defined by (31) satisfies Items (i) to (iv). $\square$

### Approximate definable choice: the case of a semialgebraic map

In this section we prove Theorem B, which we restate here.

#### Theorem 45

For every $c, d, \ell \in \mathbb {N}$ there exists $\beta >1$ satisfying the following statement. Let $n\in \mathbb {N}$ and let $K\subset \mathbb {R}^n$ be a closed semialgebraic set contained in the ball $B_{\mathbb {R}^n}(\rho )$, for some $\rho >0$, and let $F:\mathbb {R}^n\rightarrow \mathbb {R}^\ell$ be a locally Lipschitz semialgebraic map such that

$$\begin{aligned} D(\textrm{graph}(F|_K)) = (n+\ell ,c,d). \end{aligned}$$

Then for every $\epsilon \in (0, \rho )$ there exists a closed semialgebraic set $C_\epsilon \subset \mathbb {R}^{n}$ such that:

(i) $$\dim (C_\epsilon )\le \ell $$;
(ii) $$C_\epsilon \subseteq \mathcal {U}_\epsilon (K)$$;
(iii) $$\textrm{dist}_{H}(F(C_\epsilon ), F(K))\le L(F, \rho ) \cdot \epsilon $$, where $$L(F, \rho ):=2+\textrm{Lip}(F, B_{\mathbb {R}^n}(2 \rho ))$$;
(iv) for every $e=1, \ldots , n$ and every affine space $\mathbb {R}^e\simeq E\subseteq \mathbb {R}^n$, the number of connected components of $E\cap C_\epsilon$ is bounded by
  $$\begin{aligned} b_0(E\cap C_{\epsilon })\le \beta ^e. \end{aligned}$$

#### Proof

Let $\mathbb {R}^{n+\ell }=\mathbb {R}^n\times \mathbb {R}^\ell$ and denote by $\pi _1:\mathbb {R}^{n+\ell }\rightarrow \mathbb {R}^n$ and $\pi _2:\mathbb {R}^{n+\ell }\rightarrow \mathbb {R}^\ell$ the projections on the two factors.

Given $\epsilon >0$, let $A_\epsilon \subset \mathbb {R}^{n+\ell }$ be the semialgebraic set obtained by applying Theorem 44 to $S = \textrm{graph}(F|_K) \subset \mathbb {R}^{n+\ell }$, noting that by assumption the latter is a bounded and closed semialgebraic set with $D(S) = (n+\ell ,c,d)$. We define

$$\begin{aligned} C_\epsilon :=\pi _1(A_\epsilon ). \end{aligned}$$

This set is semialgebraic and closed (it is a continuous image of a compact semialgebraic set).

We verify that the set $C_\epsilon$ has the desired properties.

(i) By Item (i) of Theorem 44, $\dim (A_\epsilon )\le \ell$. Therefore, by Item (5), $\dim (C_\epsilon )\le \ell$.
(ii) Let $x\in C_\epsilon$. Then, by Item (ii) of Theorem 44, there exists *y* such that $x=\pi _1(x, y)$ with $(x, y)\in A_\epsilon \subseteq \mathcal {U}_\epsilon (S)$. Therefore there exists $(x', y')\in S$ such that
  $$\begin{aligned} \Vert x-x'\Vert +\Vert y-y'\Vert \le \epsilon . \end{aligned}$$
  This implies that $\Vert x-x'\Vert \le \epsilon$ and, since $x'=\pi _1(x', y')\in \pi _1(S)=K$, we get $x\in \mathcal {U}_\epsilon (K)$. Since this is true for all $x\in C_\epsilon$, then $C_\epsilon \subseteq \mathcal {U}_\epsilon (K)$.
(iii) Recall that we have set $L(F, \rho )=2 + \textrm{Lip}(F, B_{\mathbb {R}^n}(2\rho ))=:L$. We need to prove the two inclusions $F(C_\epsilon )\subseteq \mathcal {U}_{L\epsilon }(F(K))$ and $F(K)\subseteq \mathcal {U}_{L\epsilon }(F(C_\epsilon ))$.
  We first prove that $F(C_\epsilon )\subseteq \mathcal {U}_{L\epsilon }(F(K))$. Pick $c \in C_{\epsilon }$, then there exists $s \in A_{\epsilon }$ such that $c= \pi _1 (s)$. Since $A_{\epsilon } \subseteq \mathcal {U}_{\epsilon } (S)$ (by Item (ii) of Theorem 44), there exists $z \in S$ such that $\left\Vert z - s\right\Vert \le \epsilon$. Now, $\pi _1(z) \in K$ and we have $\left\Vert c - \pi _1(z)\right\Vert = \left\Vert \pi _1(s) - \pi _1(z)\right\Vert \le \left\Vert s - z\right\Vert \le \epsilon$. Hence, for $\epsilon \in (0,\rho )$ we have
  $$\begin{aligned} \left\Vert F(c) - F(\pi _1(z))\right\Vert&\le \textrm{Lip}(F, B_{\mathbb {R}^n}(2\rho )) \left\Vert c - \pi _1(z)\right\Vert \\&\le \textrm{Lip}(F, B_{\mathbb {R}^n}(2\rho )) \epsilon \le L(F, \rho ) \epsilon . \end{aligned}$$
  This proves that $F(C_{\epsilon })\subseteq \mathcal {U}_{L\epsilon }(F(K))$. To prove the other inclusion we fix $v \in K$. By Item (iii) of Theorem 44 we have
  $$\begin{aligned} \textrm{dist}_{H}(\pi _2(A_\epsilon ), F(K))\le \epsilon . \end{aligned}$$
  Hence, there exists $(x,y) \in A_{\epsilon }$ such that $\left\Vert F(v) - \pi _2((x, y)) \right\Vert = \left\Vert F(v) - y \right\Vert \le \epsilon$. We observe that for $\epsilon \in (0,\rho )$ we have $x \in C_{\epsilon } \subseteq \mathcal {U}_{\epsilon }(K) \subseteq B_{\mathbb {R}^n}(2\rho )$. To conclude the proof we estimate $\left\Vert F(v) - F(x)\right\Vert$. Since $A_{\epsilon } \subseteq \mathcal {U}_{\epsilon } (S)$, we find $(w, F(w) ) \in S$ such that $\left\Vert (x,y) - (w, F(w))\right\Vert \le \epsilon$, and in particular this implies $\left\Vert y - F(w)\right\Vert \le \epsilon$ and $\left\Vert x - w\right\Vert \le \epsilon$. Hence for $\epsilon \in (0,\rho )$ we have
  $$\begin{aligned} \left\Vert F(v) - F(x)\right\Vert&\le \left\Vert F(v) - y\right\Vert + \left\Vert y - F(w)\right\Vert + \left\Vert F(w) - F(x)\right\Vert \\&\le ( 2 + \textrm{Lip}(F , B_{\mathbb {R}^n}(2\rho )) ) \epsilon =L(F, \rho )\epsilon . \end{aligned}$$
  This proves that $F(K)\subseteq \mathcal {U}_{L\epsilon }(F(C_\epsilon ))$.
(iv) Let now $e\in \mathbb {N}$ with $e\le n$ and $E \simeq \mathbb {R}^e\subseteq \mathbb {R}^n$. Observe first that $E\cap C_\epsilon$ is the projection on $E\simeq \mathbb {R}^e$ of $(E \times \mathbb {R}^\ell )\cap A_\epsilon$.
  Note that $D((E\times \mathbb {R}^\ell )\cap A_\epsilon )=(e+\ell ,\kappa , \kappa d)$, where $\kappa =\kappa (c)$ is the constant of Item (iv) from Theorem 44. Therefore, the Thom–Milnor bound (8) yields
  $$\begin{aligned} b_0(E\cap C_\epsilon )\le b_0((E \times \mathbb {R}^\ell )\cap A_\epsilon )\le \beta _{\textrm{tm}}(2\kappa )(\kappa d)^{e+\ell } \le \beta (c, d, \ell )^e, \end{aligned}$$
  for suitable $$\beta =\beta (c,d,\ell )>1$$.

The proof is concluded. $\square$

## Data Availability

Data sharing not applicable to this article as no datasets were generated or analysed during the current study.
